# DNA methyltransferase 1 deficiency improves macrophage motility and wound healing by ameliorating cholesterol accumulation

**DOI:** 10.1038/s41536-023-00306-2

**Published:** 2023-06-08

**Authors:** Chuanrong Zhao, Qianru Yang, Runze Tang, Wang Li, Jin Wang, Fangfang Yang, Jianan Zhao, Juanjuan Zhu, Wei Pang, Ning Li, Xu Zhang, Xiao Yu Tian, Weijuan Yao, Jing Zhou

**Affiliations:** 1 Department of Physiology and Pathophysiology, School of Basic Medical Sciences, Peking University, Beijing, 100191 China; 2 State Key Laboratory of Vascular Homeostasis and Remodeling, Peking University, Beijing, 100191 China; 3 National Health Commission Key Laboratory of Cardiovascular Molecular Biology and Regulatory Peptides; Beijing Key Laboratory of Cardiovascular Receptors Research, Peking University, Beijing, 100191 China; 4 School of Engineering Sciences, University of Chinese Academy of Science, Beijing, 100190 China; 5 Center for Biomechanics and Bioengineering, Beijing Key Laboratory of Engineered Construction and Mechanobiology and Key Laboratory of Microgravity (National Microgravity Laboratory), Institute of Mechanics, Chinese Academy of Sciences, Beijing, 100190 China; 6 Tianjin Key Laboratory of Metabolic Diseases, The Province and Ministry Co-sponsored Collaborative Innovation Center for Medical Epigenetics, Center for Cardiovascular Diseases, Research Center of Basic Medical Sciences, Department of Physiology and Pathophysiology, Tianjin Medical University, Tianjin, 300070 China; 7 School of Biomedical Sciences, Heart and Vascular Institute, CUHK Shenzhen Research Institute, The Chinese University of Hong Kong, Hong Kong, 999077 China

**Keywords:** Chemotaxis, DNA methylation

## Abstract

Healing of the cutaneous wound requires macrophage recruitment at the sites of injury, where chemotactic migration of macrophages toward the wound is regulated by local inflammation. Recent studies suggest a positive contribution of DNA methyltransferase 1 (Dnmt1) to macrophage pro-informatory responses; however, its role in regulating macrophage motility remains unknown. In this study, myeloid-specific depletion of Dnmt1 in mice promoted cutaneous wound healing and de-suppressed the lipopolysaccharides (LPS)-inhibited macrophage motility. Dnmt1 inhibition in macrophages eliminated the LPS-stimulated changes in cellular mechanical properties in terms of elasticity and viscoelasticity. LPS increased the cellular accumulation of cholesterol in a Dnmt1-depedent manner; cholesterol content determined cellular stiffness and motility. Lipidomic analysis indicated that Dnmt1 inhibition altered the cellular lipid homeostasis, probably through down-regulating the expression of cluster of differentiation 36 CD36 (facilitating lipid influx) and up-regulating the expression of ATP-binding cassette transporter ABCA1 (mediating lipid efflux) and sterol O-acyltransferase 1 SOAT1 (also named ACAT1, catalyzing the esterification of cholesterol). Our study revealed a Dnmt1-dependent epigenetic mechanism in the control of macrophage mechanical properties and the related chemotactic motility, indicating Dnmt1 as both a marker of diseases and a potential target of therapeutic intervention for wound healing.

## Introduction

Directional chemotactic motility, i.e., migration of macrophage toward wounding or inflammatory signals, plays a critical role in many physiological and pathological reactions, including inflammation, wound healing, cancer, and formation of atherosclerotic lesions. In the early phage of cutaneous wound healing, fibrin degradation products from the clotting cascade are released from platelet degranulation, and a locally produced pro-inflammatory chemotactic gradient favors monocyte/macrophage recruitment^1^. Macrophage migration and infiltration into wounds are essential for efficient repair, as macrophages are capable of dampening inflammation, clearing cell debris and promoting cell proliferation to facilitate tissue regeneration^2^. Depletion studies have indicated that an absence of macrophages in cutaneous wound healing resulted in minimized scar formation and severe hemorrhage^3,4^. The migratory capacity of macrophages toward chemoattractant differs depending on their activation status^5^. Activation of macrophages by pathogen-associated molecules, such as lipopolysaccharide (LPS), can evoke a pro-inflammatory phenotype and inhibit their chemotactic activity^6^. While it has been recognized that the migration capacity of pro-inflammatory macrophages is weaker in comparison to the un-activated and alternatively activated (anti-inflammatory) macrophages^5,7^, the mechanisms regulating macrophage migration in inflammation are poorly understood.

Studies in mesenchymal stem cells, liver cancer stem cells, B-lymphocytes, and other cell types have suggested that stiffness (designated as elastic modulus or Young’s modulus), a crucial mechanical property of the cell, is closely related to cell motility^8–11^. Generally, a higher cell motility is correlated with lower stiffness, as cell softening favors cell deformation during migration^11^. However, recent study provided seemingly conflicting evidence showing that breast cancer cells with excellent motility were stiff^12^. So far, the correlation between cell stiffness and motility remains a matter of debate and the conclusions may depend on the cell type and methodology for the measurements. Despite intensive studies in cancer and stem cells, the correlation has rarely been studied in macrophages. In comparison, the link between macrophage stiffness and the inflammatory conditions has been established. Pro-inflammatory interferon gamma (IFNγ) has been shown to increase the cellular elastic modulus, as well as the viscous modulus, in human peripheral macrophages^13^. LPS exposure increased the elastic moduli of RAW 264.7 macrophages, suggesting that the cells became much stiffer^14^. Since pro-inflammatory stimuli inhibit macrophage migration, does the stimulated macrophage compromise its migration ability via turning stiffer?

Cholesterol is an essential component of mammalian cell membranes and the overall intracellular cholesterol distribution varies depending on cell type but mostly enriches in the plasma membrane (40–90% of the total cellular cholesterol)^15,16^. In monocytes/macrophages, the plasma membrane cholesterol counts for over 85% of the total cellular content^17^. Membrane cholesterol loading and depletion could be achieved by incubating the cells with cholesterol-chelated methyl-β-cyclodextrin (MβCD-Chol) or methyl-β-cyclodextrin (MβCD) to enable studying the influence of cholesterol homeostasis on monocyte/macrophage behaviors^18,19^. Elevated cholesterol level in the plasma membranes of monocytes/macrophages was found to increase cell spreading and inhibit migration, whereas cholesterol depletion exhibited an opposite effect^18,19^. The underlying mechanisms have been suggested to involve the regulation of actin cytoskeleton organization and actin-dependent functions, RhoA activation and the phosphorylation of myosin light chain^18,19^. Several attempts have been made to study the physical properties of plasma membranes in terms of elastic and viscous moduli affected by membrane cholesterol in red blood cells, mesenchymal stem cells, vascular smooth muscle cells, and other types of cells^20–22^, but have not been extended to monocytes/macrophages. The cholesterol-dependent modulation of plasma membrane stiffness may help to interpret the differential regulation of cellular cholesterol content on macrophage migration, which warrants further investigations.

Cholesterol homeostasis in macrophage is a complex process that depends on cooperation of a set of proteins. Macrophages take up low-density lipoproteins (LDL), very low-density lipoprotein (VLDL) and oxidized lipoproteins through macropinocytosis, phagocytosis and scavenger receptor-mediated pathways via the lectin-like oxidized LDL receptor-1 (LOX1), the class A1 and B1 scavenger receptors (SR‐A1 and SR-B1) and the cluster of differentiation 36 (CD36)^23,24^. Free cholesterol (FC) is generated in the lysosome following degradation of the taken-up lipoproteins and can be transported to the endoplasmic reticulum to be esterified by the sterol O-acyltransferase 1 (SOAT1/ACAT1). Such cholesteryl ester (CE) generated by SOAT1 is stored in cytoplasmic lipid droplets, whereas the FC is transported to the plasma membrane^23,24^. The ATP-binding cassette transporters ABCA1 and ABCG1 are responsible for as much as 70% of the total cellular cholesterol efflux for macrophage to serum or high-density lipoproteins (HDL)^25^. Despite that the regulatory mechanisms of the expression of CD36, SOAT1, and ABCA1 have been extensively studied, promoter hypermethylation and the consequent expression repression was reported in ABCA1 and SOAT1^26,27^, hinting at the involvement of the DNA methyltransferases (Dnmts) mediated epigenetic regulation on macrophage lipid homeostasis.

Temporal and spatial regulation of transcription by DNA methylation has been shown to play an important role in the epigenetic regulation of cellular function in health and disease^28^. Maintenance of methylation patterns during cell replication is mediated by DNA methyltransferase 1 (DNMT1), which catalyzes the transfer of a methyl group from S-adenosyl methionine to hemi-methylated DNA^29^. We have previously identified a positive contribution of Dnmt1 to macrophage pro-inflammatory activation and atherosclerosis^30^. In the current study, we aimed to extend the research to the investigation of whether depletion of Dnmt1 can accelerate wound healing and promote macrophage motility. Assessments of Dnmt1 regulation on cellular stiffness, lipid accumulation and cholesterol profiling, and functionality were carried out.

## Results

### Depletion of macrophage Dnmt1 accelerates cutaneous wound healing and facilitates macrophage recruitment

**Fig. 1:**
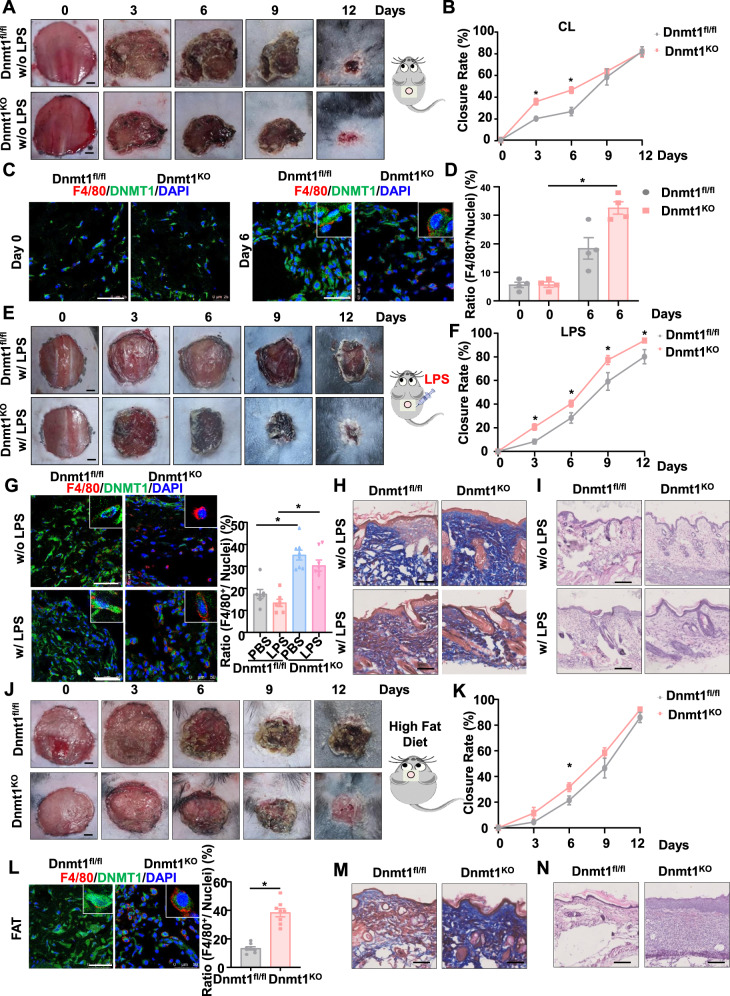
Depletion of macrophage Dnmt1 accelerates cutaneous wound healing and facilitates macrophage recruitment. **A** Representative photographs of the wounds in Dnmt1^KO^ and Dnmt1^fl/fl^ mice on day 0, 3, 6, 9, and 12 post-injury. Scale bar, 1 mm. **B** Quantification of wound closure in (**A**). The wound areas at day 0 were set as 100%, and the closure rate represents the current wound area relative to the original area (*n* = 7 for Dnmt1^fl/fl^ mice, *n* = 8 for Dnmt1^KO^ mice). **C** Immunofluorescence staining to indicate macrophage infiltration in the wounds on day 0 and day 6. Red, F4/80^+^ macrophage; Green, DNMT1; Blue, the nucleus. Scale bar, 50 μm. **D** Statistics of the ratio of the number of F4/80^+^ cells in each field to the total number of cells (DAPI). Each dot represents the average ratio from five fields of view for each mouse. **E** Representative photographs of the wounds in Dnmt1^KO^ and Dnmt1^fl/fl^ mice that have been pretreated with LPS, on day 0, 3, 6, 9, and 12 post-injury. Scale bar, 1 mm. **F** Quantification of wound closure in (**E**) (*n* = 8 for Dnmt1^fl/fl^ mice, *n* = 9 for Dnmt1^KO^ mice). **G** Immunofluorescence staining to indicate macrophage infiltration in the wounds on day 6. Red, F4/80^+^ macrophage; Green, DNMT1; Blue, the nucleus. Scale bar, 50 μm. **H**, **I** Masson (**H**) and H&E (**I**) staining of the wounds from (**G**). Scale bar, 100 μm for (**H**), 200 μm for (**I**). **J** Representative photographs of the wounds in fat Dnmt1^KO^ and Dnmt1^fl/fl^ mice on day 0, 3, 6, 9, and 12 post-injury. Scale bar, 1 mm. **K** Quantification of wound closure in (**J**) (*n* = 10 for each group). **L**–**N** Immunofluorescence (**L**), Masson (**M**), and H&E (**N**) staining of the wounds from (**J**) on day 6. Red, F4/80^+^ macrophage; Green, DNMT1; Blue, the nucleus. Scale bar, 50 μm for (**L**), 100 μm for (**M**), 200 μm for (**N**). Data are presented as mean ± SEM. *P < 0.05 by unpaired Student’s *t*-test, or two-way ANOVA followed by Sidak’s post hoc test.

Macrophage recruitment plays essential roles in cutaneous repair after mechanical injury^2,3^. To understand the role of macrophage-expressing Dnmt1 in excisional wound healing, excision wounds were made in the dorsal skin of myeloid-specific Dnmt1 knockout (Dnmt1^KO^) and Dnmt1^fl/fl^ mice^30^ (Fig. 1A). Dnmt1 expression in peritoneal macrophages from Dnmt1^KO^ mice was significantly reduced (Supplementary Fig. 1). Planimetric analysis revealed faster wound closure in Dnmt1^KO^ mice on day 3 and day 6 (Fig. 1B), suggesting that macrophage-specific Dnmt1 deficiency accelerates wound healing. During the initial phase of wound healing, myeloid cells such as macrophages, neutrophils, and monocyte-derived dendritic cells are abundant^31^. To determine the proportion of monocytes, neutrophils, and macrophages at the site of injury, live single cells were selected and analyzed using flow cytometry based on the broad myeloid cell marker CD11b, macrophage marker F4/80, and neutrophil marker Ly6G. Our analysis showed that the number and proportion of macrophages at the wound site of Dnmt1^KO^ mice exceeded that of Dnmt1^fl/fl^ mice during the early stages of the repair process (Supplementary Fig. 2). Immunofluorescent staining of wound tissues harvested on day 0 and day 6 after wounding showed increased Dnmt1-negative macrophage (F4/80^+^) infiltration on day 6 in the Dnmt1^KO^ mice (Fig. 1C, D), further suggestive of the importance of Dnmt1 in macrophage recruitment during the middle stage of cutaneous repair. As a component of microbial cell walls, LPS has been widely used to mimic systemic and local infection and to induce acute inflammation in cutaneous wound repair^32^. To assess the effect of macrophage Dnmt1 on wound healing in LPS-induced inflammation, Dnmt1^KO^ and Dnmt1^fl/fl^ mice were subcutaneously injected with LPS (10 μg/mouse) at 24 h prior to excisional wounding. We observed significantly accelerated healing in the Dnmt1^KO^ mice compared with Dnmt1^fl/fl^ mice (Fig. 1E, F). A marked increase of the presence of Dnmt1^−^ and F4/80^+^ cells was also observed in the wounds in either the LPS un-treated or treated mice on day 6 after wounding (Fig. 1G), corresponding to the planimetric data. Trichrome staining of the wounds on the same day showed increased collagen content in the granulation tissue of the Dnmt1 ^KO^ mice (Fig. 1H). Histological comparison by H&E staining of the wounds further confirmed improved wound closure in Dnmt1 ^KO^ mice (Fig. 1I). Obesity is accompanied by the development of chronic low-grade inflammation, resulting in exacerbation and persistence of inflammation and impaired tissue repair^33^. To assess the effect of macrophage Dnmt1 on wound healing in obesity-associated chronic inflammation, Dnmt1^KO^ and Dnmt1^fl/fl^ mice were fed with 60 kcal% fat diet for 5 months before the operation. We also observed significantly accelerated healing in Dnmt1^KO^ mice compared with Dnmt1^fl/fl^ mice on day 6 (Fig. 1J, K). LPS or high fat diet could significantly reduce the efficiency of wound healing on day 3, and Dnmt1^KO^ mice can partially restored the ability of wound healing (Supplementary Fig. 3). Increases of F4/80^+^ cells and collagen content in the granulation tissue of Dnmt1 ^KO^ mice were also observed in the wounds on day 6 (Fig. 1L, M). Consistently, H&E staining of the wounds on day 6 indicated improved wound closure in Dnmt1^KO^ mice (Fig. 1N). Collectively, these results demonstrate that depletion of macrophage Dnmt1 accelerate the early wound healing response. The cellular mechanisms may involve the effects of Dnmt1 depletion on macrophage recruitment to the wounds.

### Inhibition of Dnmt1 de-suppresses the LPS-inhibited macrophage motility in vitro

**Fig. 2:**
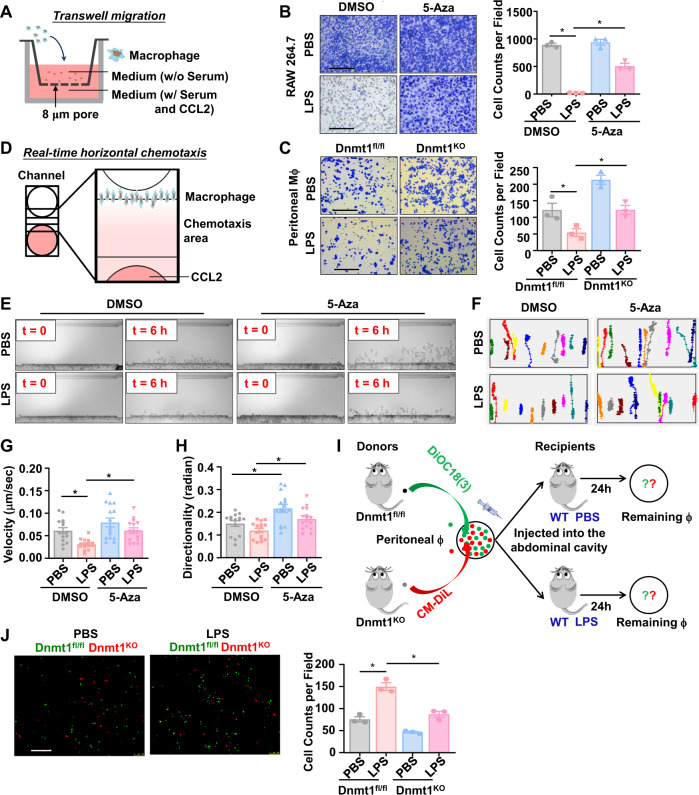
Inhibition of Dnmt1 de-suppresses the LPS-inhibited macrophage motility. **A** A schematic diagram of the transwell migration assay. **B** Transwell migration assay in RAW 264.7 cells that have been sequentially treated with 5-Aza/DMSO and LPS/PBS. Migrating cells in the outer surface of the inserts are shown by crystal violet staining. Quantitation of migrating cells was taken from the average of five fields in independent experiment (*n* = 3). Scale bar, 200 μm. **C** Transwell migration assay in peritoneal macrophages (treated with LPS/PBS) obtained from Dnmt1^fl/fl^ and Dnmt1^KO^ mice (*n* = 3). Scale bar, 200 μm. **D** A schematic diagram of the real-time horizontal chemotaxis assay. **E** Representative images taken (at time 0 and 6-h) from the real-time horizontal chemotaxis assay. Before the assay, RAW 264.7 cells were sequentially treated with 5-Aza/DMSO and LPS/PBS. **F** Representative trajectories of 10 migrating cells from the real-time horizontal chemotaxis assay. **G** Quantitation of the velocity of cell migration. Each dot means a representative cell from three independent experiments. **H** Quantitation of the directionality of cell migration. Each dot means a representative cell from three independent experiments. **I** A schematic diagram of the bead labeling migration assay performed in mice. **J** Representative images of CM-Dil (red) and DiOC_18_(3) (green) labeled peritoneal macrophages from the Dnmt1^KO^ and the Dnmt1^fl/fl^ mice recovered from the peritoneal cavity 24 h after injection of PBS or LPS into WT recipient mice. Each dot represents the average cell number of five fields of view for each mouse. Scale bar, 200 μm. Data are presented as mean ± SEM. *P < 0.05 by one-way ANOVA followed by Turkey’s post hoc test.

To explore the importance of Dnmt1 in regulating macrophage chemotactic motility in vitro, in some experiments, RAW 264.7 cells were treated with a Dnmts inhibitor 5-aza-2’-deoxycytidine (5-Aza, 10 μmol/L) for 24 h prior to an incubation with or without LPS (100 ng/ml) for 24 h, and in others, isolated peritoneal macrophages from Dnmt1 ^KO^ and Dnmt1^fl/fl^ mice were also incubated with or without LPS. Noted that LPS induced the expression of DNMT1, DNMT3A, DNMT3B and global 5-methylcytosine level in RAW 264. Seven cells (Supplementary Fig. 4), while 5-Aza could suppress all the inductions (Supplementary Fig. 5). Transwell migration assays were performed in 24 mm transwell plates with 8 μm-pore inserts (Fig. 2A). LPS significantly decreased the migration of RAW264.7 cells or peritoneal macrophages responding to serum (10%) and CCL2 (20 ng/ml), toward the lower surface of the polycarbonate membrane through the pores (Fig. 2B, C), in line with previous report^6^. Pretreatment with 5-Aza, but not the control reagent DMSO, attenuated the LPS inhibition on the chemotactic response (Fig. 2B). This effect was also observed in Dnmt1^KO^ macrophages, which exhibited normal chemotactic behavior even if a pre-exposure to LPS was present (Fig. 2C). The transwell migration assay cannot exclude the influences of gravitational force thus we employed a real-time horizontal chemotaxis assay using TAXIScan (Fig. 2D). Direct comparison of the first (0 h) and the last (6 h) frames from the recorded video of the chemotaxis of RAW 264.7 cells toward CCL2 (20 ng/ml) identified suppression of LPS on the cell chemotactic responses and a de-suppressive effect of Dnmt1 inhibition by the treatment with 5-Aza (Fig. 2E). The cells were manually tracked to show the movement trajectories (Fig. 2F). The obtained images by 6 h were subjected to quantification for directionality and velocity (Fig. 2G, H). The CCL2-stimulated macrophages migrated toward the other end of the channel where the concentration of CCL2 was the highest (Fig. 2F), indicative of directional rather than spontaneous motility. For most of the tracked cells, LPS seemed to decrease the migration distances, and the decreases could be ameliorated by Dnmt1 inhibition (Fig. 2F). The directionality and velocity in response to CCL2 were reduced by the treatment with LPS; the reduction could be avoided in the presence of 5-Aza pre-treatment (Fig. 2G, H). Results from the horizontal chemotaxis assay using the Dnmt1^KO^ and the Dnmt1^fl/fl^ macrophages verified that LPS inhibited the chemotactic responses in Dnmt1^fl/fl^ cells and that the impairment in their chemotactic motility could be rescued by Dnmt1 depletion (Supplementary Fig. 6). The findings suggest that Dnmt1 is involved in negatively regulating chemotactic motility of LPS-primed pro-inflammatory macrophages.

### Depletion of Dnmt1 promotes macrophage motility in a bead labeling migration assay

To investigate the migration behavior of Dnmt1^KO^ macrophages in vivo, we utilized a bead labeling migration assay^34^. In this assay, equal amounts of CM-Dil (chloromethyl-dialkylcarbocyanine, red) and DiOC_18_(3) (3,3-Dioctadecyloxacarbocyanine perchlorate, green) labeled peritoneal macrophages from the Dnmt1^KO^ or Dnmt1^fl/fl^ mice were injected into the peritoneal cavity of wild type (WT) recipients, accompanied by intraperitoneal injection with LPS (10 μg/mouse) or PBS, and 24 h later, the amount of retained labeled macrophages was measured (Fig. 2I). Similar amounts of Dnmt1^fl/fl^-green and Dnmt1^KO^-red macrophages were demonstrated before injection, indicative of equal initial cell quantities (Supplementary Fig. 7). At 24 h post-injection, peritoneal macrophages were isolated from the recipients. A 1.98-fold increase in the numbers of labeled Dnmt1^fl/fl^ cells remained in the peritoneal cavity of the LPS-treated mice, compared with that in the PBS-treated mice, indicating impairment in migration of Dnmt1^fl/fl^ macrophage by LPS treatment (Fig. 2J). Depletion of Dnmt1 significantly decreased the numbers of retained labeled cells, demonstrative of a profound improvement in migration compared to the Dnmt1^fl/fl^ macrophages (Fig. 2J). These results suggest that depletion of Dnmt1 de-suppresses the LPS-inhibited macrophage motility in vivo.

### Inhibition of Dnmt1 eliminates the LPS-stimulated cellular stiffening

**Fig. 3:**
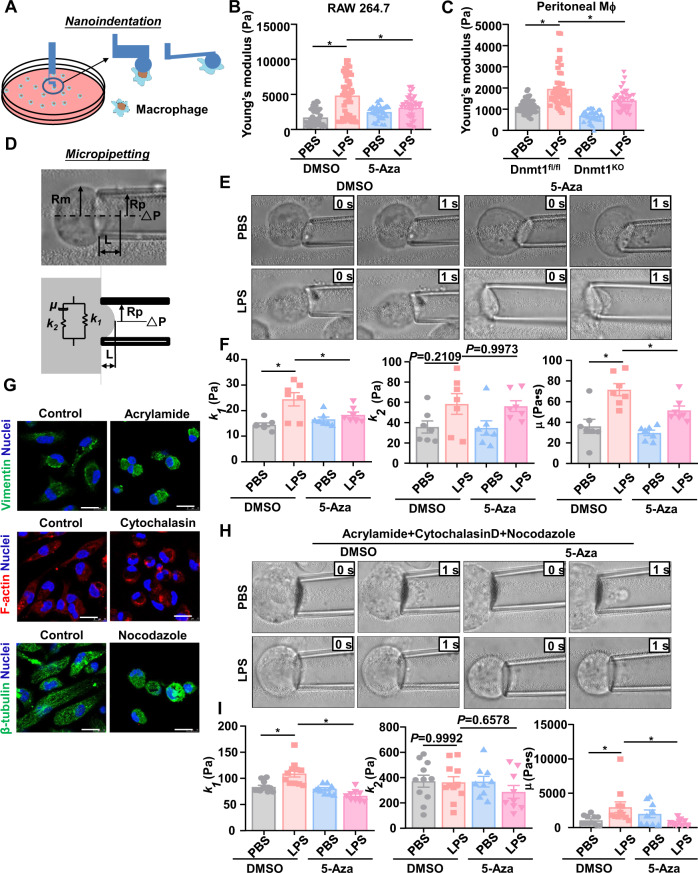
Inhibition of Dnmt1 eliminates the LPS-stimulated cellular stiffening. **A** A schematic diagram of the nanoindentation test to measure the cell stiffness. **B**, **C** The cellular stiffness measured by nanoindentation. In (**B**), RAW 264.7 cells were sequentially treated with 5-Aza/DMSO and LPS/PBS. Each dot means a representative cell from five independent experiments. In (**C**), peritoneal macrophages from Dnmt1^KO^ and Dnmt1^fl/fl^ mice were treated with LPS/PBS. Each dot means a representative cell from 3 to 5 mice. **D** A schematic diagram of the micropipette aspiration test to measure the cell viscoelasticity. **E** Representative brightfield images of RAW 264.7 cells immediately before (0 s) and after (1 s) partial aspiration into a micropipette. The cells were treated as in (**B**). **F** The viscoelastic parameters (*k*_1_, *k*_2_ and *μ*) of RAW 264.7 cells from (**E**). Each dot represents a single cell. Data were obtained from 3–5 independent experiments. **G** Immunofluorescence staining of cytoskeletons in RAW264.7 cells treated with cytoskeleton depolymerization compounds (acrylamide, cytochalasin, or nocodazole), respectively. Scale bar, 20 μm. **H** Representative brightfield images of RAW 264.7 cells immediately before (0 s) and after (1 s) partial aspiration into a micropipette. The cells were sequentially treated with cytoskeleton depolymerization compounds, 5-Aza/DMSO, and LPS/PBS. **I** The viscoelastic parameters (*k*_1_, *k*_2_ and *μ*) of RAW 264.7 cells from (**H**). Each dot represents a single cell. Data were obtained from 3-5 independent experiments and are presented as mean ± SEM. *P < 0.05 by one-way ANOVA followed by Turkey’s post hoc test.

Characterization of the physical properties of macrophages has discovered topographic and mechanical changes upon LPS stimulation^14,35^. We then sought to investigate whether Dnmt1 would affect the cellular mechanics of macrophages under an LPS-primed pro-inflammatory status. We first determined the Young’s moduli by a nanoindentation technique in (1), 5-Aza- and DMSO-treated RAW 264.7 cells, and (2), Dnmt1 ^KO^ and Dnmt1^fl/fl^ peritoneal macrophages, in the presence or absence of LPS treatment for 24 h (Fig. 3A). The overall Young’s moduli were 1721 $\pm \,$1132 and 1113 $\pm \,$31 Pa for the control RAW 264.7 and peritoneal macrophages, respectively; the cells became much stiffer (4838 $\pm \,$2902 and 1963 $\pm \,$88 Pa) than the control cells after LPS exposure (Fig. 3B, C). The LPS-induced increases were attenuated by Dnmt1 inhibition (Fig. 3B, C), suggestive of a role of Dnmt1 in mediating the LPS-stimulated cell stiffening. Cell stiffness/elasticity and viscoelasticity have been reported as important factors related to macrophage motility^13,14,35,36^. The lower the elasticity and viscoelasticity of a cell, the easier the cell deforms and migrates^13^. The mechanical property of a cell is mainly determined by its cytoskeleton, the membrane, and the nucleus^37,38^. To confirm the results from the nanoindentation experiments and to further measure cell viscoelasticity, we performed micropipette aspiration in RAW 264.7 cells (Fig. 3D). A standard linear viscoelastic solid model was employed to analyze the viscoelastic behavior of the cells, where *k*_1_ and *k*_2_ are the elastic elements and *μ* is a viscous element^39^. In comparison with the control treatment, LPS caused increases in the *k*_1_ and *μ* values, but not the *k*_2_ value; the increases were suppressed by 5-Aza treatment (Fig. 3E, F). Changes in *k*_1_ and *k*_2_ have been suggested to mainly reflect the membranal alternations or cytoskeleton re-organization, repectively^40^. The above observations suggest that LPS elevates the cellular elasticity and viscosity, in particular the membrane elasticity, via Dnmt1. To further exclude the contribution of cytoskeleton components to the LPS-induced cellular biomechanical changes, cytoskeleton-disrupting drugs were employed. The effects of treatment of macrophages with an actin polymerization inhibitor cytochalasin D, a microtubule assembly/disassembly disruptor nocodazole, and an intermediate filament disruptor acrylamide, on cytoskeleton organization were verified by immunofluorescent staining (Fig. 3G). After cytoskeleton disruption, LPS still caused increases in *k*_1_ and *μ*, compared with the control treatment (Fig. 3H, I), hinting that the LPS effects on the cellular mechanical properties are independent of influencing cytoskeleton organization. The LPS effects were further abolished by treatment of the cells with 5-Aza (Fig. 3H, I). These results demonstrate that pro-inflammatory stimuli stiffen the cells (increase their elasticity/viscoelasticity), and that this effect is mediated by Dnmt1.

### Inhibition of Dnmt1 ameliorates the LPS-induced cholesterol accumulation

**Fig. 4:**
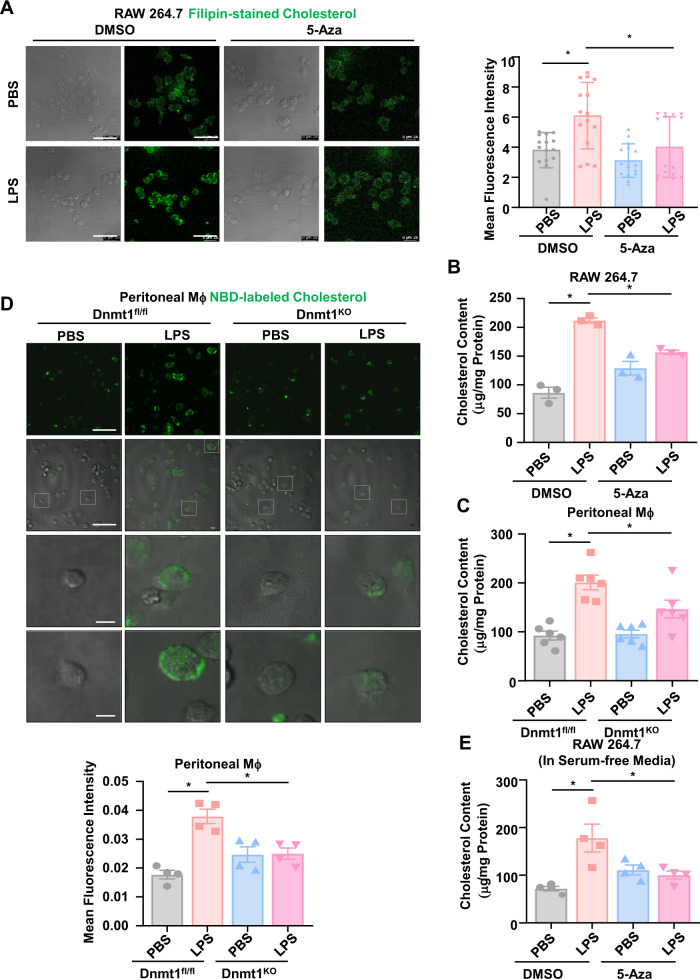
Inhibition of Dnmt1 ameliorates the LPS-induced cholesterol accumulation. **A** Filipin staining of free cholesterol in RAW 264.7 cells (*n* = 3). The cells have been sequentially treated with 5-Aza/DMSO and LPS/PBS. Scale bar, 50 μm. **B** The total cholesterol content of RAW264.7 cells that were sequentially treated with 5-Aza/DMSO and LPS/PBS (*n* = 3). **C** The total cholesterol content of peritoneal macrophages (treated with LPS/PBS) from Dnmt1^KO^ and Dnmt1^fl/fl^ mice (*n* = 6). **D** Live-cell fluorescence images showing NBD-labeled cholesterol uptake by peritoneal macrophages (treated with LPS/PBS) from Dnmt1^KO^ and Dnmt1^fl/fl^ mice. The mean fluorescence intensity was obtained for the average of five fields in independent experiment (*n* = 4). Scale bar, 50 μm for upper two lines and 10 μm for lower two lines. **E** The total cholesterol content of RAW264.7 cells that were sequentially treated with 5-Aza/DMSO and LPS/PBS in serum-free media (*n* = 4). Data are presented as mean ± SEM. *P < 0.05 by one-way ANOVA followed by Turkey’s post hoc test.

Cholesterol is an essential component of the plasma membranes, which are the structures that border the cell and maintain the cell integrity. Plasma membrane exhibits both elastic and viscous properties that are modulated by the presence of cholesterol^41^. We examined the cholesterol levels by filipin staining that recognizes free, unesterified cholesterol, in RAW 264.7 cells that were either pre-treated with 5-Aza or DMSO and then subjected to LPS or PBS incubation. LPS increased the filipin fluorescence, while Dnmt1 inhibition with 5-Aza moderated the increase (Fig. 4A). Notably, the filipin cholesterol staining was mainly observed in the plasma membranes (Fig. 4A). An enzymatic assay was used to quantitatively measure the cellular cholesterol and indicated that the total cholesterol content was induced to 2.46-fold of the control by LPS treatment in the DMSO-treated groups and that the induction was attenuated by 5-Aza (Fig. 4B). Measuring the cellular cholesterol contents in Dnmt1 ^KO^ and Dnmt1^fl/fl^ peritoneal macrophages returned the similar results (Fig. 4C). Cholesterol homeostasis in macrophages is regulated at several levels, including uptake, synthesis, storage, metabolism, and efflux^24^. The uptake of fluorescence of nitrobenzoxadiazole (NBD)-labeled cholesterol in peritoneal macrophages from Dnmt1^fl/fl^ mice was remarkably increased after LPS stimulation for 24 h, and the increase was suppressed by Dnmt1 depletion (Fig. 4D), suggesting that the LPS-induced Dnmt1 may change the cholesterol content by promoting its uptake. By using serum-free medium to block the cholesterol uptake, we further demonstrated that LPS induced cholesterol accumulation in the absence of exogenous cholesterol, and that Dnmt1 inhibition could still suppress the LPS-induced cholesterol accumulation (Fig. 4E), suggestive of an involvement of Dnmt1-regulated cholesterol homeostasis beyond the uptake level. Altogether, the LPS-induced, Dnmt1-dependent modulation on cholesterol homeostasis may be regulated at multiple levels.

### Cholesterol content determines the macrophage stiffness and motility

**Fig. 5:**
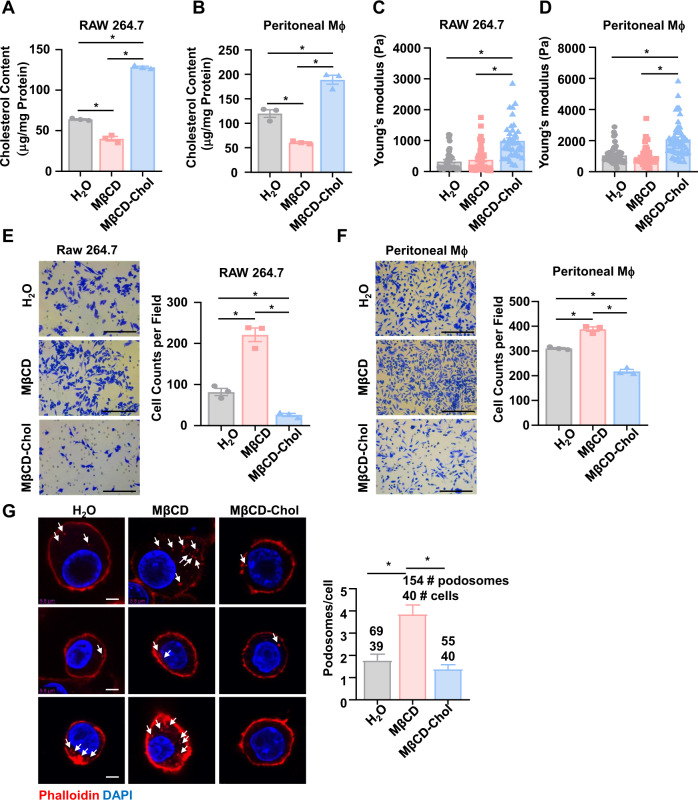
Cholesterol content determines the macrophage stiffness and motility. **A** The total cholesterol content of RAW 264.7 cells treated with MβCD or MβCD-Chol (*n* = 3). **B** The total cholesterol content of peritoneal macrophages treated with MβCD or MβCD-Chol (*n* = 3). The cells were obtained from the Dnmt1^KO^ and Dnmt1^fl/fl^ mice. **C** The cellular stiffness (measured by nanoindentation) of RAW264.7 cells treated with MβCD or MβCD-Chol. Each dot represents a single cell. **D** The cellular stiffness (measured by nanoindentation) of peritoneal macrophages treated with MβCD or MβCD-Chol. The cells were obtained from the Dnmt1^KO^ and Dnmt1^fl/fl^ mice. Each dot represents a single cell. **E** Transwell migration assay in RAW 264.7 cells treated with MβCD or MβCD-Chol. Quantitation of migrating cells was taken from the average of five fields in independent experiment (*n* = 3). Scale bar, 200 μm. **F** Transwell migration assay in peritoneal macrophages from the Dnmt1^KO^ and Dnmt1^fl/fl^ mice. The cells were treated with MβCD or MβCD-Chol. Quantitation of migrating cells was taken from the average of five fields in independent experiment (*n* = 3). Scale bar, 200 μm. **G** Representative images of the phalloidin-stained F-actin foci (red) and DAPI-stained nucleus (blue) in RAW264.7 cells treated with MβCD or MβCD-Chol. Arrows indicate the podosomes. Statistics showed the number of podosomes in each cell. Scale bar, 5 μm. Data are presented as mean ± SEM. *P < 0.05 by one-way ANOVA followed by Turkey’s post hoc test.

Most of the cellular cholesterol is found in the membranes, where it increases the bilayer stiffness^42^. MβCD has a central cavity able to form a 2:1 complex with cholesterol, and thus is the most widely used reagent for acute cholesterol depletion^43^. In contrast to cholesterol depletion, treatment of cells with MβCD-Chol would increase the cholesterol content. In both RAW 264.7 and peritoneal macrophages isolated from the Dnmt1^fl/fl^ mice, the cholesterol contents were decreased by MβCD (10 mmol/L) to 37.2% and 49.3%, and increased by MβCD-Chol (0.5 mmol/L) by 100.5% and 57.6%, respectively (Fig. 5A, B). In parallel experiments, treatment of the cells with MβCD-Chol significantly increased the cellular stiffness, while treatment with MβCD did not yield such strong effect (Fig. 5C, D), consistent with the previous report^21^. Functionally, macrophages treated with MβCD exhibited very much improved migration ability than that with the control treatment, whereas the cells treated with MβCD-Chol had compromised migration capacities (Fig. 5E, F). Macrophage migration is driven by dynamic rearrangements of the cytoskeleton, with formation of actin-based structures, podosomes, that are usually 0.5-2 μm in diameter and concentrating toward the leading edge of migrating cells^44^. Podosome formation was boosted by cholesterol depletion with MβCD in RAW 264.7, indicating that lower cholesterol content is correlated with higher migration capacity (Fig. 5G). The findings verify a central role of the cellular cholesterol content in determining the macrophage stiffness and motility.

### Dnmt1 regulates the cellular cholesterol homeostasis

**Fig. 6:**
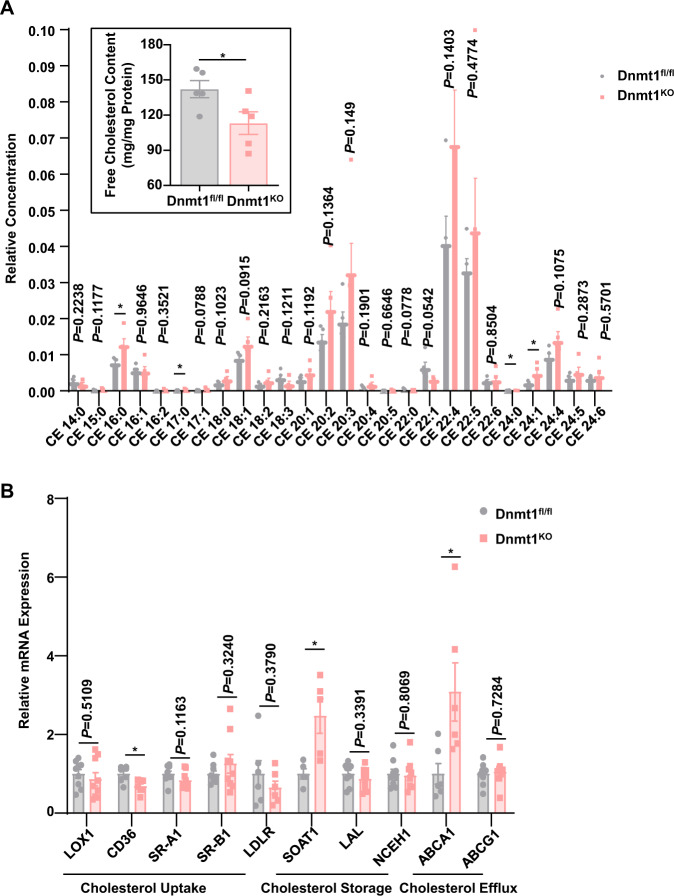
Dnmt1 regulates the cellular cholesterol homeostasis. **A** The upper left insert shows the content of free cholesterol in peritoneal macrophages from the Dnmt1^KO^ and Dnmt1^fl/fl^ mice. The contents of cholesteryl ester in peritoneal macrophages from the Dnmt1^KO^ and Dnmt1^fl/fl^ mice were measured by UHPLC-MS. **B** Real-time qPCR analysis of expression of key genes in cholesterol uptake, esterification and efflux in peritoneal macrophages. The cells were obtained from the Dnmt1^KO^ and Dnmt1^fl/fl^ mice (*n* = 5–7). Data are presented as mean ± SEM. *P < 0.05 by unpaired Student’s t-test.

Macrophage cholesterol homeostasis is maintained not only by a balance between the influx and efflux pathways, but also by a conversion between biologically active FCs and inactive CEs, the latter may function as a biologically inert form of cholesterol for storage^45^. The total cholesterol contents of Dnmt1^KO^ and Dnmt1^fl/fl^ peritoneal macrophages were quite similar (Supplementary Fig. 8 and Fig. 4C); however, assessment of the CE constitute and content using an ultrahigh performance liquid chromatography-mass spectrometry (UHPLC-MS) method identified significant increases of CE 16:0, CE 17:0, CE 24:0, and CE 24:1 in the Dnmt1^KO^ peritoneal macrophages, compared to that in the Dnmt1^fl/fl^ macrophages (Fig. 6A). The FC contents also differed: the Dnmt1-depleted macrophages had remarkably lower FC content than the control cells (Fig. 6A). These results indicate that Dnmt1 might positively regulate the conversion of cholesterol from CEs to FCs. We conducted RNA sequencing of peritoneal macrophages from DNMT1^fl/fl^ and DNMT1^KO^ mice to provide insight into the overall impact of Dnmt1 deficiency on gene expression and have identified 2,750 genes with changes in expression (|log2FoldChange| ≥ 0.2), of which 117 were enriched in lipid metabolism based on Gene Ontology (GO) analysis (Supplementary Fig. 9). The expressions of ABCA1, SOAT1, and CD36, which we had focused on, were consistent with our in vitro findings. To understand how Dnmt1 regulates macrophage cholesterol homeostasis, we detected a set of genes responsible for cholesterol synthesis, uptake, storage, or efflux, in Dnmt1^KO^ and Dnmt1^fl/fl^ peritoneal macrophages (Supplementary Fig. 10 and Fig. 6B). No significant difference of the expression of genes related to cholesterol synthesis was discovered (Supplementary Fig. 10). Oxidized cholesterol (oxLDL) influx into macrophage is facilitated by several scavenger receptors including LOX1, CD36, SR-A1, SR-B1, and the LDL receptor (LDLR)^24^. In the endoplasmic reticulum, SOAT1 catalyzes esterification of FCs to CEs to store them as lipid droplets^46^. In the cytoplasm and the lysosomes, the neutral cholesterol ester hydrolase 1 (NCEH1) or the lysosomal acid lipase (LAL) hydrolyzes CEs to FCs, respectively^24^. FC could be then out‐flowed by cholesterol ATP‐binding cassette (ABC) transporters ABCA1 and ABCG1^25^. In the Dnmt1^KO^ peritoneal macrophage, CD36 was significantly downregulated, while SOAT1 and ABCA1 were upregulated, Compared with that in the control cells, (Fig. 6B), in line with a less intracellular FC and a boosted FC-to-CE conversion in the KO cells (Fig. 6A). Collectively, these findings demonstrate that Dnmt1 is involved in the regulation of macrophage cholesterol homeostasis and the mechanism may involve its transcriptional regulation on CD36, SOAT1 and ABCA1.

### Dnmt1 regulates cholesterol homeostasis and cellular stiffness by mediating ABCA1 and SOAT1 methylation

**Fig. 7:**
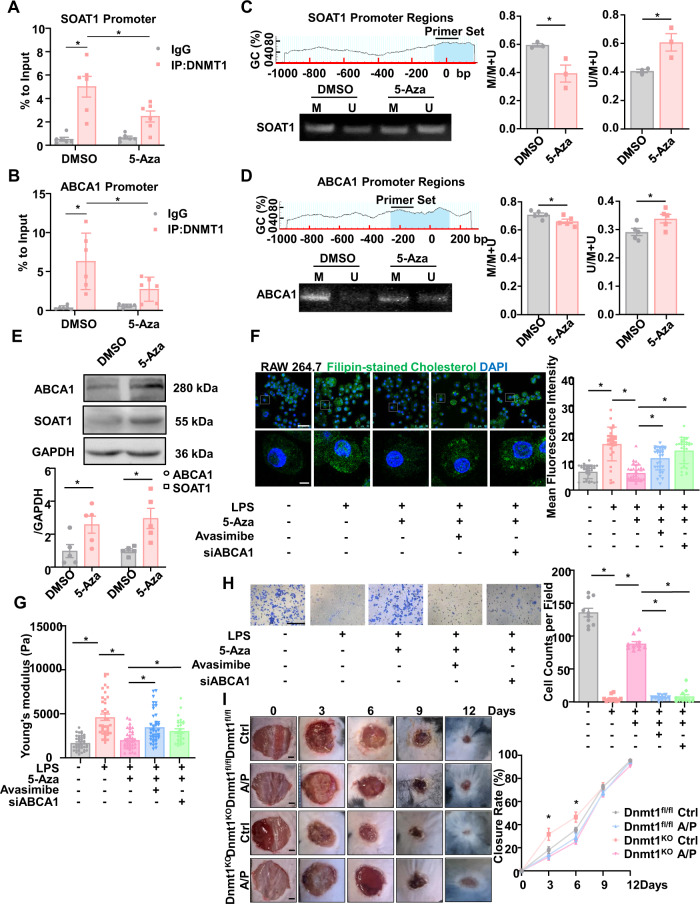
Dnmt1 regulates cholesterol homeostasis and cellular stiffness by mediating ABCA1 and SOAT1 methylation. ChIP assay to detect the binding of Dnmt1 to the promoter regions of SOAT1 (**A**) and ABCA1 (**B**) (*n* = 6). MSP assay to detect the methylation status of SOAT1 (**C**) and ABCA1 (**D**) in RAW264.7 cells treated with DMSO/5-Aza. The CpG islands in the promoter regions of SOAT1 or ABCA1 were indicated by blue shade. Statistics shows the proportion of methylation band (M) or unmethylation band (U) to the total population (*n* = 3 or 5). **E** Western blotting and the quantification to indicate the protein levels of SOAT1 and ABCA1 in RAW264.7 cells treated with DMSO/5-Aza (*n* = 5). **F** Filipin staining of cholesterol in RAW264.7 cells treated with DMSO/5-Aza, DMSO/avasimibe (an inhibitor of SOAT1), siRNA targeting ABCA1 (siABCA1), and PBS/LPS. Scale bar, 50 μm for upper line and 10 μm for lower line. **G** The cellular stiffness (measured by nanoindentation) of RAW264.7 cells treated with DMSO/5-Aza, DMSO/avasimibe, control siRNA/siABCA1 and PBS/LPS. **H** Transwell migration assay in RAW 264.7 cells treated with DMSO/5-Aza, DMSO/avasimibe, control siRNA/siABCA1 and PBS/LPS. Quantitation of migrating cells was taken from each field. Scale bar, 200 μm. **I** Representative photographs of the wounds in Dnmt1^KO^ and Dnmt1^fl/fl^ mice that have been treated with control PF-127 or avasimibe and probucol (A/P) in PF-127, on day 0, 3, 6, 9, and 12 post-injury. Scale bar, 1 mm. Quantification of wound closure rate in four group, * means *P* < 0.05 between Dnmt1^KO^-Ctrl with other three groups (*n* = 7 for each group). Data are presented as mean ± SEM. *P < 0.05 by unpaired Student’s *t*-test, one- or two-way ANOVA followed by Turkey’s post hoc test or Sidak’s post hoc test.

Dnmt1 binding and the Dnmt1-mediated DNA methylation at the promoter regions are usually associated with gene silencing or transcriptional repression^47^. We assessed the Dnmt1 binding to the ABCA1 or SOAT1 promoters, as the expression of both genes was increased by Dnmt1 depletion (Fig. 6B). Chromatin immunoprecipitation indicated that, relative to the control IgG, Dnmt1 was highly enriched in the SOAT1 and ABCA1 promoters in the RAW 264.7 macrophages, and that treatment with 5-Aza reduced the enrichment (Fig. 7A, B). The promoter regions of SOAT1 and ABCA1 contain the typical cytosine-phosphate-guanine (CpG) islands with a guanine-cytosine (GC) percentage >50%, therefore are ideal for methylation assessment using a methylation-specific polymerase chain reaction (PCR) (MSP) assay, that indicated hypermethylation of the promoters in the DMSO-treated cells (Fig. 7C, D). Inhibition of Dnmt1 with 5-Aza decreased the methylation levels in the both promoters (Fig. 7C, D). Accordingly, the protein levels of SOAT1 and ABCA1 were raised by 5-Aza (Fig. 7E). No CpG island was found in the promoter regions of CD36 (Supplementary Fig. 11A). Although Western blotting indicated that 5-Aza decreased the CD36 expression (Supplementary Fig. 11B), the mechanism is unlikely to be based solely on promoter-methylation.

Lastly, we sought to answer whether Dnmt1 inhibition softens macrophage by the mediation of SOAT1 and ABCA1. Avasimibe that inhibits SOAT1 activity and CE synthesis, and the small interfering RNA targeting ABCA1 (siABCA1) were employed as the loss-of-function approaches. The efficiency of siRNA-mediated knockdown of ABCA1 was demonstrated by real-time qPCR (Supplementary Fig. 12). Filipin cholesterol staining revealed that the LPS-induced cellular cholesterol accumulation was suppressed by 5-Aza, and that the accumulation could be regenerated by treatment of the cells with avasimibe or transfection with siABCA1 (Fig. 7F). Measurement of the cell stiffness by nanoindentation indicated that cell stiffening upon LPS stimulation was inhibited by 5-Aza, and the stiffening effect could be reproduced by treatment with avasimibe or by ABCA1 knockdown (Fig. 7G), echoing the changes in the cellular cholesterol content. Rescue experiments of transwell migration assay with avasimibe and siABCA1 in DMSO or 5-Aza treated cells in the presence or absence of LPS further demonstrated the importance of the DNMT1-SOAT1/ABCA1 axis in mediating the LPS-induced cellular response (Fig. 7H). To link our in vitro results with our in vivo findings, we encapsulated two inhibitors, avasimibe^48^ and probucol^49^, in a thermosensitive PF-127 hydrogel^50^ and applied it to a full-thickness cutaneous wound to inhibit SOAT1 and ABCA1 respectively. Representative images of the wound area in each group on days 0, 3, 6, 9, and 12 after surgery are presented (Fig. 7I). Planimetric analysis showed faster wound closure in Dnmt1^KO^-control mice on day 3 and day 6 than in the other three groups. Combined treatment with avasimibe and probucol inhibited the local wound closure rate of Dnmt1^KO^ mice but had no significant impact on Dnmt1^fl/fl^ mice, indicating the significance of the DNMT1-SOAT1/ABCA1 axis in macrophage recruitment during the early stage of cutaneous repair.

## Discussion

It is well known that macrophages have dampening inflammation and clearing cell debris capabilities and play a key role in promoting healing of the cutaneous wounds^51^. Despite a prior study that indicated a hematopoietic stem cell (HSC)-expressing Dnmt1 represses the differentiation of HSCs toward macrophages/monocytes and therefore impairs cutaneous wound healing^52^, the role of macrophage Dnmt1 in influencing wound healing has not been reported elsewhere. In this study, myeloid-specific Dnmt1 depletion enhanced wound repair, as evidenced by faster wound closure and elevated collagen deposition. These effects are in agree with the pro-inflammatory role of Dnmt1 in macrophages as reported previously by us and others^30,53,54^, implying that insufficient Dnmt1 expression might assist with wound healing by ameliorating the pro-inflammatory microenvironment surrounding the wounds. Moreover, it has been recognized that efficient migration of macrophages to the injured sites for activating the adaptive immune response through cytokine production and antigen-presentation is required for cutaneous wound healing^55^. Our study also indicated an increased number of macrophages in the wound sites in the Dnmt1 ^KO^ mice, suggesting that a myeloid-expressing of Dnmt1 might hinder the chemotactic migration of macrophages. To further verify the role of Dnmt1 in regulating the chemotactic migration of macrophages, we have conducted in vitro and/or in vivo studies to assess migration of the WT/control and the Dnmt1-depleted/-inhibited macrophages in response to the chemotactic stimuli, serum and a chemokine CCL2. We also observed that knockout or inhibition of Dnmt1 enhanced macrophage migration, in line with the in vivo wound healing assay. In terms of regulating cell migration, the role of Dnmt1 differs and depends on the cell types, which might be related to the various mechanisms. For instance, in human mesenchymal stem cells, their migration ability was improved after Dnmt1 inhibition^56^; whereas in choriocarcinoma cells or gastric cancer cells, Dnmt1 inhibition decreased their migration^57,58^. Our study provides strong evidence demonstrating a negative contribution of Dnmt1 to the chemotactic migration of macrophages, in particular in an obesity/LPS-primed pro-inflammatory status.

It is intriguing to note that the mechanical properties of cells change during inflammation. For example, hypertension and aging-imposed pro-inflammatory stresses augment vascular smooth muscle cell stiffness^59^; anti-inflammatory glucocorticoids such as dexamethasone soften leukocytes^60^. Specifically, in monocytes, THP-1-derived macrophages, and RAW 264.7 macrophages, the inflammation status has been found positively correlated with the cellular stiffness^14,61–63^. Echoing the previous study, our study found that treatment of cells with LPS increased their Young’s modulus, the elastic modulus *k*_1_ (but not *k*_2_), and the viscous modulus *μ* of macrophages, suggesting a phenotype of cellular stiffening. More importantly, we discovered that the LPS-induced macrophage stiffening is mediated by Dnmt1, as evidenced by the characterization of mechanical properties in cells with Dnmt1 loss-of-function. On the basis of the above results and prior findings that, for example, in rat bone marrow-derived mesenchymal stem cells and B lymphocytes cell, increased cell stiffness was often accompanied by decreased cell migration^8,10^, the effect of Dnmt1 on limiting macrophage migration capacity may be explained by its ability to stiffen the cells. Furthermore, the effects of Dnmt1 on cell stiffness might be specified to altering the mechanical properties of the membrane, as in the presence of cytoskeleton disruption reagents, the Dnmt1-dependent regulation of the cellular mechanical properties by LPS still occurred.

Cholesterol is essentially confined to plasma membranes in mammalian cells, being involved in regulating the permeability barrier properties of lipid bilayers and affecting the membrane fluidity^41^. Needham D et al. have reported that an increase in cellular cholesterol content in the engineered cell membranes could improve the elastic compression modulus of the membranes and increase the cell stiffness^20^. In the study, we verified the correlation between membrane cholesterol content and cellular stiffness by using a classical approach for adding or subtracting membrane-cholesterol. Our results indicated that adding cholesterol with incubation with MβCD-Chol increased the cell stiffness. Likely as a consequence, podosome formation and chemotactic migration were inhibited accordingly. Although the differences in the Young’s modulus of the control and MβCD-treated cell were undetectable, functional studies demonstrated increased cell migration and podosome formation in MβCD-treated macrophages versus that in the control. In addition, we observed that LPS modified the cholesterol loading to the membrane in a Dnmt1-dependent manner. LPS has been reported to affect DNA methylation by activating active DNA demethylation processes via the tet methylcytosine dioxygenases and/or thymine DNA glycosylase enzymes^64^, thus a DNA demethylation-mediated mechanism might also be involved in the LPS-induced cellular epigenetic regulation. Loss-of-function study by Dnmt1 depletion/inhibition demonstrated a predominant role of Dnmt1. Given these, our findings robustly suggest that LPS induced Dnmt1 to cause more cholesterol accumulation to the membranes to stiffen the cells. Nevertheless, one should not ignore the contradictions that, such as, in bovine aortic endothelial cells and human embryonic kidney cells, lowering cholesterol increased the cell stiffness by mechanisms that may be related to affecting cytoskeleton F-actin^65,66^. When the cellular cholesterol increases, F-actin may be proportionally increased and distribute in bundles in the cytoplasm, leading to the reduction in the cell stiffness^67^.

With the utilize of lipidomic analysis to quantitatively investigate the CE components in this study, it was interesting to observed that, compared with the WT macrophages, Dnmt1 depletion decreased the FC content and increased the CEs. Furthermore, gene expression analysis indicated that CD36 was decreased by Dnmt1 depletion, whereas SOAT1 and ABCA1 were increased. Based on the above results, we have been able to propose and finally, validate that Dnmt1 influences a number of pathways that have been previously implicated in regulating cholesterol homeostasis (e.g., influx, esterification, and efflux). Our findings are consistent with prior studies in which the expressions of CD36, SOAT1 and ABCA1were regulated by the Dnmt1-mediated promoter methylation in various types of cells or tissues^26,68,69^. Specifically, we provided evidence demonstrating the roles Dnmt1-SOTA1 and Dnmt1-ABCA1 cascades in mediating the LPS-stimulated cholesterol accumulation and cell stiffening, with the usage of Dnmt1 inhibitor 5-Aza, SOAT1 inhibitor avasimibe, and siABCA1.

**Fig. 8:**
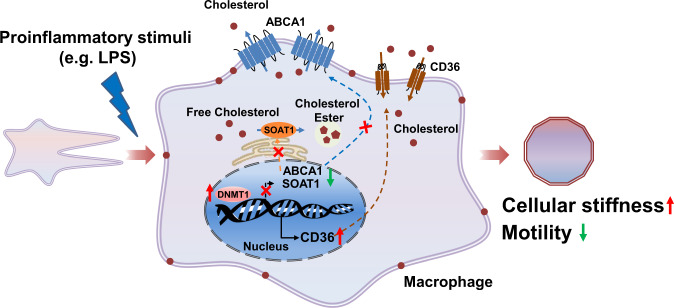
DNA methyltransferases 1 (Dnmt1) regulates macrophage chemotactic motility and cellular stiffness that are modified by inflammation. Release of the pro-inflammatory LPS upon bacterial infection of the wounds may induce the expression of Dnmt1, which in turn upregulates the expression of CD36 and downregulates the expression of SOAT1 and ABCA1, probably by a DNA-methylation-based mechanism. The increased CD36 and decreased SOAT1/ABCA1 levels cause alterations in the cellular cholesterol homeostasis, leading to cholesterol accumulation in the plasma membranes to stiffen the cells and consequently to limit cell motility.

Overall, the present study demonstrates a critical role of macrophage Dnmt1 in dampening cutaneous wound repair and macrophage recruitment to the injury sites. Release of the pro-inflammatory LPS upon bacterial infection of the wounds may induce the expression of Dnmt1, which in turn upregulates the expression of CD36 and downregulates the expression of SOAT1 and ABCA1, probably by a DNA-methylation-based mechanism. The increased CD36 and decreased SOAT1/ABCA1 levels cause alterations in the cellular cholesterol homeostasis, leading to cholesterol accumulation in the plasma membranes to stiffen the cells and consequently to limit cell motility (Fig. 8).

## Methods

### Experimental animals

All animal studies were performed in accordance with the guidelines of the Animal Care and Use Committee of Peking University and approved by the Ethics Committee of Peking University Health Science Center (LA2015017). Mice were kept in specific pathogen-free cages, 12 h light-dark cycle, controlled temperature and humidity, and had water and food ad libitum. The methods of obtaining the Dnmt1^KO^ and the Dnmt1^fl/fl^ mice have been described in the previous work of our laboratory^30^.

### Cell culture

RAW 264.7 cells (the murine-derived macrophage cell line, ScienCell, XY-XB-1204, introduction from ATCC TIB-71) were cultured in high-glucose Dulbecco’s modified Eagle’s medium (DMEM) containing 10% fetal bovine serum in a humidified incubator with 5% CO_2_ at 37 °C. Macrophages were treated with 5-Aza (Selleck, China) in culture medium at a concentration of 10 μmoL/L for 24 h to achieve DNMT1 inhibition. The cells were incubated with LPS (Sigma Aldrich, Merck, Germany) at a concentration of 100 ng/ml for 24 h to induce inflammation. In order to increase or decrease the content of cellular cholesterol, macrophages were treated with 0.5 mmol/L methyl-β-cyclodextrin-cholesterol (Sigma, USA) or 10 mmol/L methyl-β-cyclodextrin (Sigma, USA) for 1 h at 37 °C. To achieve loss-of-function of SOAT1 and ABCA1, RAW 264.7 cells were treated with 10 µmol/L Avasimibe (SOAT1 inhibitor) for 24 h or transfected with siRNA targeting ABCA1 (sense-5′-GUGUCUACGUGCAACAGAU-3′, antisense-5′-AUCUGUUGCACGUAGACAC-3′).

To isolate mouse peritoneal macrophages, 10-week-old Dnmt1^fl/fl^ and Dnmt1^KO^ mice were injected intraperitoneally with 2 ml of 4 % sterile thioglycolate broth at 3 days before the operation. The mice were properly euthanized and soaked in 75% ethanol for 5 min. The mice were then fixed with the abdomen upward and the first layer abdominal skin of the mice was carefully removed. Then 8 ml of sterile PBS containing 3% fetal bovine serum cooled in advance was injected. The abdomen was massaged for 5 min and the peritoneal lavage fluid were recovered. Peritoneal macrophages were obtained by centrifugation and then cultured in RPMI-1640 Medium containing 10% fetal bovine serum in a humidified incubator with 5% CO_2_ at 37 °C.

### Wound healing assay

Aged-matched, randomly assigned mice were anesthetized by 1.5-2% isoflurane, and hair-removal-cream was used for removing the hair on the back of mice. After disinfecting the exposed skin, a circle with a diameter of 10 mm was drawn on the back of the mouse, and the full layer of skin in this area was removed. The removed skin tissue was soaked in 4% paraformaldehyde (PFA) for subsequent experiments. A steel ruler was placed next to the skin wound, then photos of the wound were taken on day 0, 3, 6, 9 and 12. The areas of the wound were calculated with ImageJ software. To assess the effect of macrophage Dnmt1 on wound healing in LPS-induced acute inflammation, Dnmt1^KO^ and Dnmt1^fl/fl^ mice were subcutaneously injected with LPS (10 μg/mouse) 24 h prior to excisional wounding^32^. To assess the effect of macrophage Dnmt1 on wound healing in fat-induced chronic inflammation, Dnmt1^KO^ and Dnmt1^fl/fl^ mice were fed with 60 kcal% fat diet (Research Diets, USA) for 5 months before the operation. To demonstrate the role of SOAT1 and ABCA1 in wound healing, avasimibe (a SOAT1 inhibitor, 0.1 mg/ml, Yuanye, S80444) and probucol (an ABCA1 inhibitor, 0.1 mg/ml, Aladdin, P129365) were encapsulated in a thermosensitive PF-127 hydrogel (24%, 100 μl/per wound, Sigma, P2443) to a full-thickness cutaneous wound every three days. The mice were euthanized by carbon dioxide inhalation. Briefly, adult mice were placed into the euthanasia induction chamber (PS-0661, Rothacher Medical GmbH, Switzerland), compressed CO_2_ gas with a fill rate of 30–70% of the chamber volume per minute add added to the existing air in the chamber. Mice were unconsciousness usually within 2 to 3 min. Maintain CO_2_ flow for a minimum of 1 min after respiration ceases. Then the euthanasia of mice was confirmed by an ascertaining cardiac and respiratory arrest.

### Dissociation of wound tissue and flow cytometry analysis

Wound samples were intensely cut into small pieces using surgical scissors. After washed with PBS, samples were resuspended in 2 mL RPMI 1640 medium and predigested using mixed enzyme solution (0.2 mg/ml collagenase IV, 1 mg/ml trypsin) treatment at 37 °C and shaking at 65 rpm for 20 min. Neutralized with serum solution and re-digested with mixed enzyme solution. Repeat three times for 60 min. The cell suspension was diluted with 1× PBS and passed through a 100 μm cell strainer (Biosharp, BS-100). Cells were pelleted by centrifugation, transferred to an EP tube. They were washed with PBS and stained for 30 min in PBS containing a dye that selectively stains viable cells (live/dead Fixable Blue Dead Cell Stain Kit, Invitrogen, 2559165) and antibodies directed against different cell surface markers (FITC anti-mouse F4/80 recombinant, BioLegend 157309, 1:100; PE/Cyanine7 anti-mouse Ly-6G, BioLegend 127617,1:100; PE anti-mouse/human CD11b, BioLegend 101207, 1:100) Following cell surface staining, cells were washed with PBS, fixed with 4% PFA for 20 min and washed once with permeabilization buffer (saponin). For intracellular staining, DNMT1 antibody (Abcam, ab188453, 1:100) was diluted in permeabilization buffer and cells were stained overnight. They were then washed with PBS, incubated with Goat Anti-Rabbit IgG H&L (Alexa Fluor 647, Abcam, ab150083, 1:200) for 1 h, and stored at 4 °C in the dark until acquisition. Stained cells were analyzed using a BD LSRII Fortessa (BD Biosciences). Compensation adjustment, gating, and data analysis were performed using FlowJo software (Version X, Tree Star Inc., Ashland, OR), and data were exported for further processing.

### Immunofluorescence (IF) staining

Tissue sections were fixed in 4% PFA for 15 min and permeabilized with 0.25% Triton X-100 in PBS for another 15 min. After being blocked with 3% bovine serum albumin (BSA) in PBS for 1 h, sections were probed with F4/80 (BioLegend, 123101, 1:100) primary antibody overnight. Then, sections were washed and probed with Goat Anti-Rat IgG H&L (Alexa Fluor 568, Abcam, ab175476, 1:300) for 2 h. Nuclei were counterstained with DAPI for 5 min. Images of the stained samples were captured with Leica TCS-SP8 and Leica TCS-SP8 DIVE (Leica Microsystems, Wetzlar, Germany).

### Histological analysis

Skins of wound healing site were collected at 6 days after the operation. The embedded tissues were sliced into 7-μm-thick sections. Hematoxylin and eosin (H&E) staining was used to evaluate the morphological structure of the skins. Masson staining was applied according to the manufacturer’s instructions (60532ES74, Yeasen, Shanghai, China). Collagen fibers appear blue and muscle fibers appear red. Images of the stained samples were captured with NanoZoomer-SQ (HAMAMATSU PHOTONICS K.K., Japan).

### Transwell migration assay

Transwell migration assays were performed in 24 mm transwell plates with 8 μm pore inserts (Corning, USA). Cell were put into the upper chamber of the transwell inserts with no-serum medium, and complete medium with 10% serum and C-C motif chemokine 2 (CCL2) at a concentration of 20 ng/ml was added to the lower chamber. After the cells migrated for 24 h, cells on the inner of the membrane were wiped off and on the outer of the membrane were fixed by 4% PFA for 15 min and stained by crystal violet (Beyotime Biotechnology, Shanghai) for 30 min. The number of migrated cells was quantified by counting the average number of cells from 5 random fields at the microscope.

### Real-time horizontal chemotaxis assay

Chemotactic responses of macrophages were measured using the real-time chemotaxis assay device, TAXIScan (ECI, Japan). Silicon chips with a 10-μm-high terrace were used, which forms a 10-μm-deep microchannel. After filling the space at the top end of the holes with RPMI-1640, 10 μl of macrophage suspensions (1 × 10^5^ cells/ml) were injected into one of the two compartments, and the cells were aligned along the start line on the edge of the channel. 5 μl of CCL2 (20 ng/ml) was injected into the compartment opposite that containing the cells. Cellular migration at 37 °C was recorded using a camera focused on the migration path every 3 min for 1–6 h. The velocity of cell migration and the trajectory of cells were analyzed using TAXIScan analyzer 2 software.

### In vivo migration assay

Equivalent fresh peritoneal macrophages were extracted from 8–12 weeks old Dnmt1^fl/fl^ and Dnmt1^KO^ mice, and incubated with cell membrane fluorescent probe DiOC_18_(3) (green, Yeasen Biotechnology, Shanghai) and CM-Dil (red, Yeasen Biotechnology, Shanghai) respectively at 37 °C for 20 min according to the manufacturer’s instructions. Then cells were mixed softly in the centrifuge tube with 220 μl of PBS, and two 100 μl of cell suspensions were injected into the abdominal cavity of two C57/BL6J wildtype (WT) mice, respectively. Some of them were injected with 100 μl of PBS, and others were injected with 100 μl of LPS (10 μg). After 24 h, peritoneal macrophages of two mice were recovered, and the number of two fluorescent labeled macrophages in the field of vision was calculated under the fluorescence microscope Leica DMI600B (Leica Microsystems, Wetzlar, Germany).

### Measurement of cell stiffness with nanoindentation

Ferruled optical fiber-based nanoindenter was used for testing cell stiffness. We used the ferrule-top nanoindenter setup together with the PIUMA controller/drive (Optics11, Amsterdam; The Netherlands). By combining fiber-optical Fabry-Perot interferometry with a monolithical cantilever-based probe, local micro-elasticity can be examined with high accuracy and precision. The spring constant of the probe is 0.048 N/m, and the spherical indentation tip is 9 μm. Macrophages were cultured on a Petri dish with the diameter of 6 cm. After the pretreatment, the medium was replaced with OPTI-MEM medium with the nanoindenter tip remaining well below the surface of buffer at all times. During indentation the tip was brought into contact with the cell surface and load-indentation and load-time data were recorded. The indents were depth controlled (5 μm) and the loading and unloading period was set to be 2 s. Based on the load-displacement curves the reduced Young’s modulus was calculated using the Hertz spherical indentation model. For cell stiffness analysis, the mean Young’s modulus was generated from 20 to 30 single measurements in three independent tests.

### Micropipette aspiration technology

Micropipette aspiration technique was adopted to measure viscoelastic properties of macrophages. The following picture (a) shows the system. A negative pressure was applied via adjusting the height of reservoir gauged by a micrometer, and the movement of macrophages inside the pipette was recorded by a charge-coupled device camera (Watec, Japan). In our experiments, the diameter of the micropipette was about 7 μm, the chamber was filled with PBS, and the pretreated macrophages were injected into the chamber. One cell was sucked into the tip of the pipette with a negative pressure given to it. A standard linear viscoelastic solid model was employed to analyze the viscoelastic behavior of macrophages and cells were regarded as a semi-infinite medium. The following formula^70^ shows the relation between aspirated lengths (L) and time of cells.:

1 $$L=\frac{2{R}_{p}\Delta P}{{\rm{\pi }}{k}_{1}}\times \left[1+\left(\frac{{k}_{1}}{{k}_{1}+{k}_{2}}-1\right){{exp}} \left(-\frac{{k}_{1}{k}_{2}t}{\mu ({k}_{1}+{k}_{2})}\right)\right]$$

Where *L, t, ΔP, Rp* are the aspirated lengths, aspirated time, aspiration pressure and micropipette radius, respectively. This model is composed of two parallel arms which is showed in the picture (b), where *k*_*1*_ and *k*_*2*_ are elastic elements, and *μ* is viscous element. The elastic elements *k*_*1*_ and *k*_*2*_, which do not possess time-dependent characteristics by themselves, determine the degree of the initial rapid deformation of the cell, which varies with [(1/*k*_*1*_) + (1/*k*_*2*_)]. The viscous element (*μ*) provides the time-dependent behavior, the combination of *μ* with the two elastic elements determines the rate of creep during the slow phase of deformation, with the time constant given by *μ* (*k*_*1*_ + *k*_*2*_)/*k*_*1*_ *k*_*2*_. The maximum deformation for long periods of time is proportional to 1/*k*_*1*_^40^. When *t* tends to infinity, *L* reaches the maximum *L*_*max*_ and the formula can be expressed as

2 $${L}_{\max }=\frac{2{R}_{p}\Delta P}{{\rm{\pi }}{k}_{1}}$$

So the result of *k*_*1*_ can be calculated. When we select different *t* and corresponding *L*, the values of *k*_*2*_ and *μ* can be obtained by the formula.

A schematic diagram of the micropipette aspiration technology and a diagram of data measurement were in the supplementary materials (Supplementary Fig. 13).

To further exclude the contribution of cytoskeleton components to the LPS-induced cellular biomechanical changes, actin polymerization inhibitor cytochalasin D (2 μmol/L), microtubule assembly/disassembly disruptor nocodazole (10 μmol/L), and intermediate filament disruptor acrylamide (4 μmol/L) were employed for 1 h, 2 h and 16 h, respectively. After treatment, micropipette aspiration technique was adopted to measure viscoelastic properties of macrophages.

### Total/Free cholesterol content measurements

An enzymatic assay was used for determining the content of total cholesterol and free cholesterol levels of cells according to the manufacturer’s instructions (Applygen Technologies, E1015 and E1016, Beijing). Briefly, cells were washed with PBS to remove the remaining serum from the culture medium and then added with lysis buffer in the kit. The working solution and cholesterol standard substance were diluted. After incubation at 37 °C for 20 min, OD values were measured at 550 nm by multimode microplate reader (Thermo Fisher, USA) to calculate cholesterol content.

### F-actin and filipin staining

Cell were seeded on cover slips for different treatment. Cover slips were fixed in 4% PFA for 15 min and permeabilized with 0.3% Triton X-100 in PBS for another 15 min at room temperature. Then cells were incubated with Rhodamine Phalloidin (ABclonal, RM02835, 1:200) for 30 min or 50 µg/mL filipin (Cayman, 70440) for 1.5 h. Nuclei were counterstained with DAPI for 5 min (for F-actin staining). Images of the stained samples were captured with Leica TCS-SP8 and Leica TCS-SP8 DIVE.

### Assessment of the uptake of exogenous NBD-labeled cholesterol

Dnmt1^KO^ and Dnmt1^fl/fl^ peritoneal macrophages were seeded in 30 mm aperture laser confocal dish. Cells were treated with LPS at 100 ng/ml for 24 h at 37 °C. The cells were then washed with PBS for three times and incubated in phenol red-free RPMI 1640 medium containing 5 µmol/L NBD-cholesterol (Cayman, 13220, USA) for 4 h at 37 °C. Cell images were captured with Leica TCS-SP8 and Leica TCS-SP8 DIVE.

### UHPLC-MS analysis for cholesterol esters

Quantification of cholesteryl esters in macrophages from Dnmt1^fl/fl^ and Dnmt1^KO^ mice were performed on a Waters Acquity UPLC system coupled to an Sciex 5500 triple-quadrupole mass spectrometer with the ESI ionsource in MRM mode. Data acquisition and processing were performed by Analyst software (version 1.5.1).

### RNA purification and real-time quantitative PCR

RNAs were extracted by using TRIzol reagent (Applygen Technologies, Beijing) according to the manufacturer’s instructions, and samples were stored at −40 °C. Complementary DNAs (cDNAs) were obtained with M-MLV RT system (Promega) by using Oligo (dT) primers. Real-time quantitative PCR (q-PCR) was performed with 2X RealStar power SYBR Mixture (Genestar) by using the specific primer pairs (below). Gene expressions were normalized against GAPDH. Primers for qPCR are listed in Supplementary Table 1.

### Transcriptome analysis

Total RNA from the Dnmt1^KO^ and Dnmt1^fl/fl^ peritoneal macrophages was extracted using the RNeasy kit with DNase treatment (Qiagen) and RNA integrity (RIN > 9) was checked by a Bioanalyzer 2100 system (Agilent Technologies). cDNA libraries were constructed using the NEBNext UltraTM RNA Library Prep Kit according to the manufacturer’s instructions (Illumina). Cluster was generated using cBot Cluster Generation System with TruSeq PE Cluster Kit v3-cBot-HS (Illumia). After cluster generation, sequencing was performed on an Illumina Novaseq platform and 150 bp paired-end reads were generated. Index of the reference genome was built using Hisat2 v2.0.5 and paired-end clean reads were aligned to the mus musculus genome using Hisat2 v2.0.5. FeatureCounts v1.5.0-p3 was used to count the reads numbers mapped to each gene. FPKM of each gene was calculated based on the length of the gene and reads count mapped to this gene. Differential expression analysis was performed using the DESeq2 R package (1.20.0). The resulting *P*-values were adjusted using the Benjamini and Hochberg’s approach for controlling the false discovery rate. In total, 2750 genes were differentially expressed in the pairwise comparisons (*n* = 2, │log_2_ Fold Change│ ≥ 0.2). Pathway over-representation analysis was performed using clusterProfiler R package to test the statistical enrichment of differential expression genes in GO database (http://geneontology.org/).

### Chromatin immunoprecipitation-polymerase chain reaction (ChIP-PCR)

RAW 264.7 cells seeded in 100 mm dishes at 10^7^ cells/dish were fixed and cross-linked with 1% formaldehyde in PBS for 10 min. Then the cells were lysed with 500 μl of lysis buffer (1% SDS, 5 mM EDTA, 50 mM Tris-HCl [pH 8.1], plus protease inhibitors), incubated on ice for 10 min, and sonicated for 15 times at 1 s each. The lysates were centrifuged at 12,000 rpm at 4 °C for 10 min. Supernatants were collected and a 20 μl aliquot was set aside as input fraction. The remaining supernatants were diluted in 1:2 with dilution buffer (1% Triton X-100, 2 mM EDTA, 150 mM NaCl, 20 mM Tris. HCl [pH 8.1], 1 X protease inhibitor cocktail) and separated equally, incubated with antibody against DNMT1 (abclonal, A16729, 1:100) or the control IgG at 4 °C overnight, and then with 20 μl of protein A-beads for another 1 h. After centrifugation at 3000 rpm for 5 min, the beads were washed sequentially with 1 ml of buffer TSE I (0.1% SDS, 1% Triton X-100, 2 mM EDTA, 20 mM Tris. HCl [pH 8.1], 150 mM NaCl), buffer TSE II (0.1% SDS, 1% Triton X-100, 2 mM EDTA, 20 mM Tris. HCl [pH 8.1], 500 mM NaCl), buffer III (0.25 M LiCl, 1% NP-40, 1% deoxycholate, 1 mM EDTA, 10 mM Tris. HCl [pH 8.1]) and TE buffer. DNAs were eluted from the beads with 100 μl of elution buffer (1% SDS, 0.1 M NaHCO_3_) at room temperature for 10 min. The eluates were heated at 65 °C overnight to reverse the formaldehyde cross-linking. DNAs were extracted with a DNA pure-spin kit (Vigorous, #N009). Finally, RT-qPCR was performed to determine the enrichment of Dnmt1 in the SOAT1 and ABCA1 promoters. Primers for ChIP-qPCR are listed in Supplementary Table 1.

### MSP

Genomic DNA of RAW 264.7 was extracted by DNA extraction buffer (50 μl TE buffer, 450 μl STE buffer, 10 μl 20% SDS,10 μl protein K (10 mg/ml)). Bisulfite modification of DNA (1 μg) was performed with the EpiMark Bisulfite Conversion Kit (NEB, USA) according to the manufacturer’s instructions. MethPrimer (http://www.urogene.org/methprimer) was used for determining the methylation status and design the primers of methylated or unmethylated sequences of ABCA1 and SOAT1. Polymerase chain reaction (PCR) amplifications were carried out in a total volume of 25 μl by using Taq DNA Polymerase (TIANGEN). MSP reactions were subjected to initial incubation at 95 °C for 5 min, followed by 40 cycles of 95 °C for 30 s, and annealing at the appropriate temperature for 30 s and 72 °C for 30 s. Final extension was done by incubation at 72 °C for 7 min. MSP products were separated on 2% agarose gels and visualized after Gel-Red (Beyotime Biotechnology, Shanghai) staining. Primers for MSP are listed in Supplementary Table 1.

### Western blotting

Cells were diluted in PBS with phenylmethylsulphonylfluoride (PMSF, 1 μmol/L) and freeze-thawed three times using liquid nitrogen. After that, the soluble fraction (lysate) was separated from the cell debris by centrifugation at 20,000 × *g* for 20 min at 4 °C. The concentration of protein was determined by the BCA (Thermo Fisher Scientific, USA) assay. Equal amounts of protein were separated on SDS-polyacrylamide gel electrophoresis (PAGE), transferred to nitrocellulose membranes, blocked with 5% skim milk in TBST, and incubated with the primary antibodies (ABCA1 (ABclonal, A16337, 1:1000), SOAT1 (ABclonal, A6311, 1:1000), CD36(ABclonal, A5792, 1:1000), DNMT1 (ABclonal, A16729, 1:1000), DNMT3A (ABclonal, A2065, 1:1000), or DNMT3B (ABclonal, A11079, 1:1000) and GAPDH (EASYBIO, BE0024, 1:1000)) overnight. After incubation with appropriate HRP-conjugated goat anti-rabbit/mouse IgG (CW0103S/CW0102S, Procell, 1:4000), immunoreactive bands were visualized by ECL (Biodragon).

### Immuno-dot blot assay

Genomic DNA of RAW 264.7 was extracted by DNA extraction buffer (50 μl TE buffer, 450 μl STE buffer,10 μl 20% SDS,10 μl protein K (10 mg/ml)). The DNA samples (2 μg per dot) were spotted on a nitrocellulose membrane in a Bio-Dot SF apparatus (Bio-Rad). The membrane was baked at 80 °C for 2 h, blocked in 5% skimmed milk in TBS containing 0.1% Tween 20, and then incubated with 1:1000 dilution of 5-meC antibody (Beyotime, AF5722) overnight at 4 °C, followed by its detection using secondary antibody. Odyssey Fluorescent imaging system (LI-COR Biosciences) was used for visualization. For the staining of total DNA, the blot membrane was hybridized with 0.2% methylene blue (Leagene, DZ0094).

### Statistical analysis

Data were expressed as mean ± standard error of the mean (SEM). Unpaired Student’s *t*-test was conducted when two groups were compared. For analysis of more than two groups, one-way ANOVA followed by Tukey’s multiple comparison test and two-way ANOVA followed by Sidak’s multiple comparison test were used for pairwise comparison between groups. *P* < 0.05 were considered as statistic significant. The software for statistical analysis is GraphPad Prism 8.0.

### Reporting summary

Further information on research design is available in the Nature Research Reporting Summary linked to this article.

## Supplementary information

Reporting Summary

Supplementary Information

## Data Availability

All data are available in the main text or the supplementary materials. All the raw sequencing data have been deposited at National Center for Biotechnology Information Gene Expression Omnibus, https://www.ncbi.nlm.nih.gov/geo (GEO ID GSE233629). All gels and blots were processed in parallel and derive from the same experiment, uncropped images were attached in Supplementary Information (Supplementary Figs. 14, 15). Data files are available from the corresponding author on reasonable request.
