# Supplementary material for: DNA methyltransferase 1 deficiency improves macrophage motility and wound healing by ameliorating cholesterol accumulation
Source: NPJ Regen Med. 2023 Jun 8;8:29. doi: 10.1038/s41536-023-00306-2 (PMC10250321; doi:10.1038/s41536-023-00306-2)
Supplement: Supplementary file 2 — Supplementary Information [file 41536_2023_306_MOESM2_ESM.pdf]

Supplementary Information

Supplementary Figures and Legends

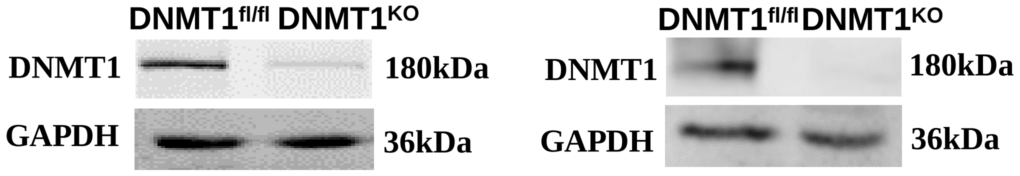

Supplementary Figure 1. The expression level of DNMT1 in peritoneal macrophages of Dnmt1^{KO} and Dnmt1$^{fl/fl}$ mice. Western blotting to detect the protein level of Dnmt1 in peritoneal macrophages of Dnmt1^{KO} and Dnmt1$^{fl/fl}$ mice (n=2).

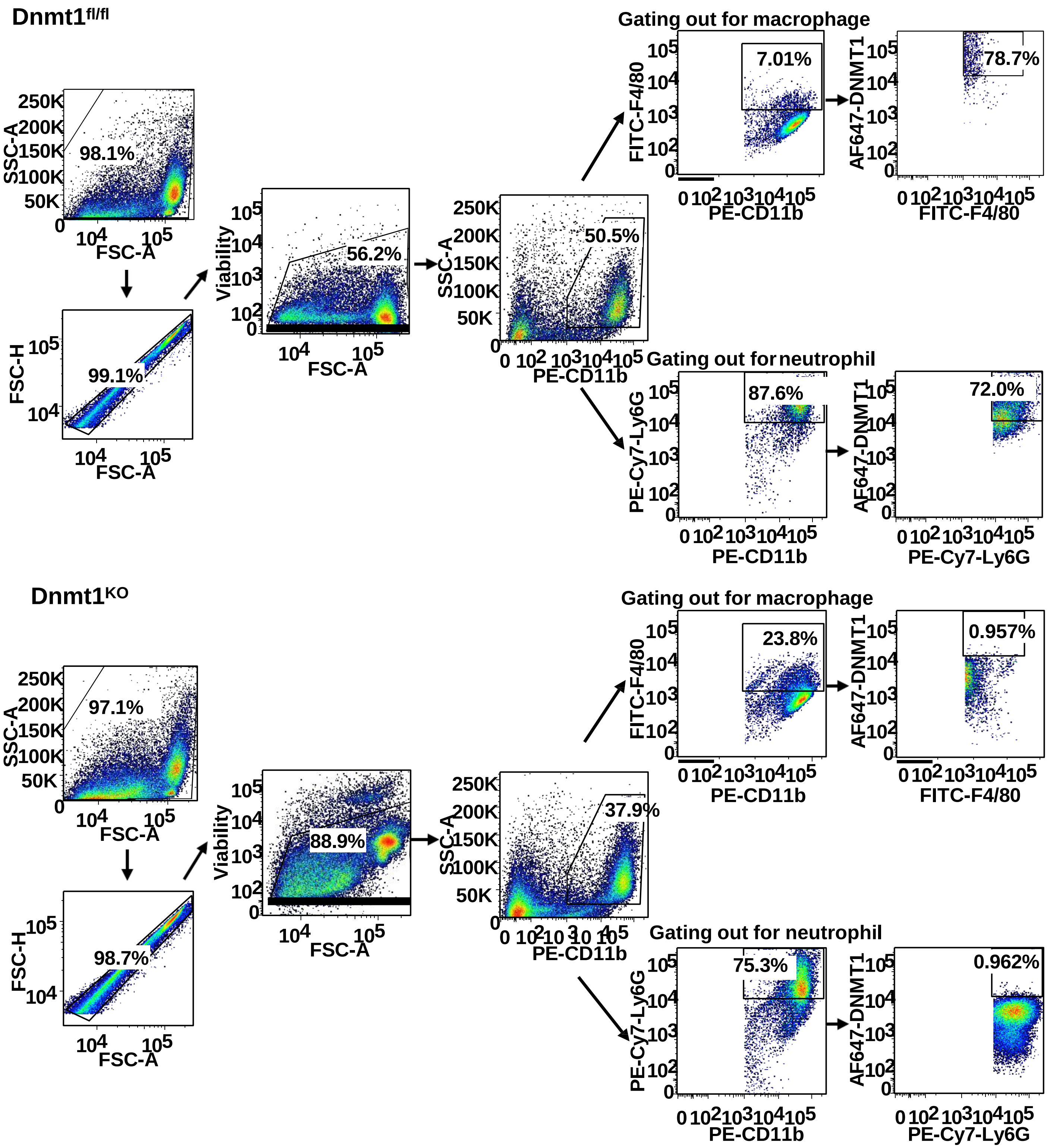

Supplementary Figure 2. Flow cytometry gating strategy for analysis myeloid cells at day 3 in wound healing of Dnmt1$^{fl/fl}$ and Dnmt1^{KO} mice. Representative flow cytometry gating plots for the analysis of myeloid cell populations. Live single cells were selected and analyzed for total myeloid cells (CD11b^{+}) based on broad myeloid cell marker CD11b. Alternatively, F4/80^{+} cells were gated as macrophages and Ly-6G^{+} cells were defined as neutrophils, the remaining cells were identified as monocytes or other monocyte-derived cells. Flow cytometry analysis showed that among the living cells isolated from the skin of DNMT1$^{fl/fl}$ mice after 3 days of wound healing, ~56.2% were CD11b^{+} myeloid cells, with ~87.6% of those cells being Ly-6G^{+} neutrophils and 7.01% being F4/80^{+} macrophages, which were ~78.7% DNMT1-positive. The remaining cells accounted for ~5.39%. In contrast, in DNMT1 myeloid-knockout (DNMT1^{KO}) mice, CD11b^{+} cells accounted for ~37.9% among the ~88.9% of living cells, with the proportion of neutrophils decreasing to ~75.3% and macrophages increasing to ~23.8%. The positive rate of DNMT1 sharply decreased to ~0.957% in macrophages and ~0.962% in neutrophils.

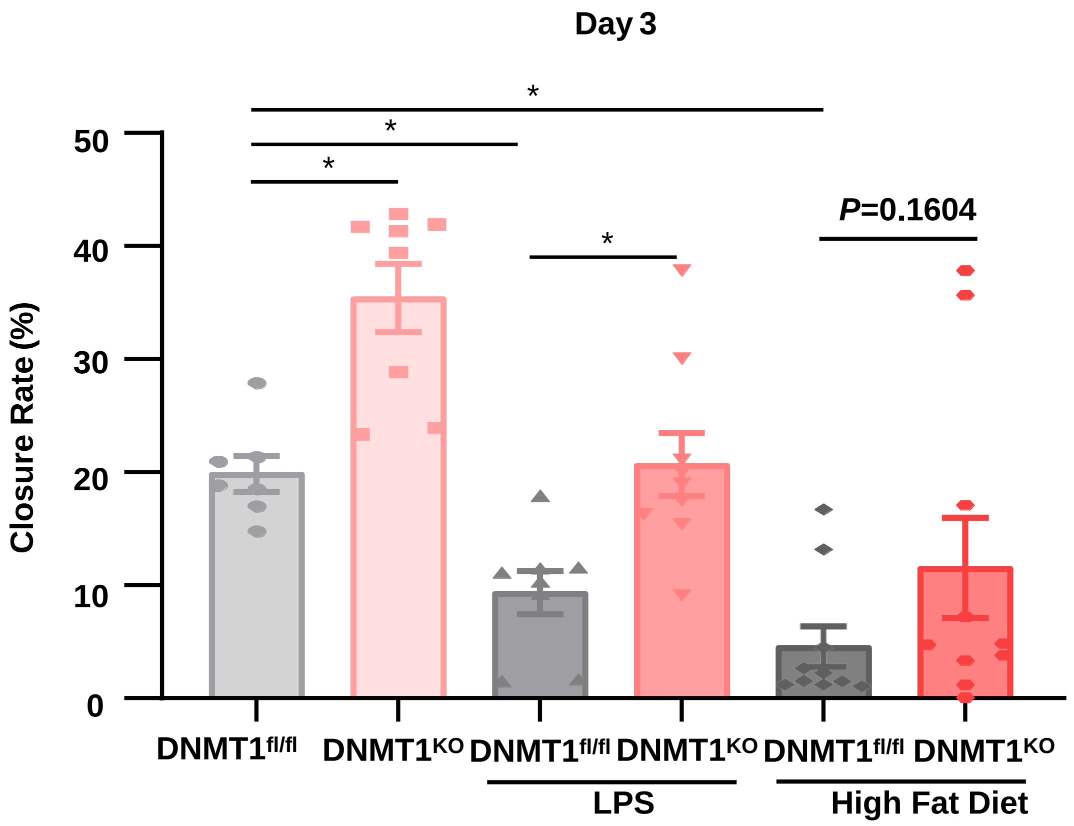

Supplementary Figure 3. Quantification of wound closure in $Dnmt1^{KO}$ and $Dnmt1^{fl/fl}$ mice on day 3. $Dnmt1^{KO}$ and $DNMT1^{fl/fl}$ mice were no treated (n=7 for $Dnmt1^{fl/fl}$ mice, n=8 for $Dnmt1^{KO}$ mice), or subcutaneously injected with LPS (10 µg/mouse) at 24 h prior to excisional wounding (n=8 for $Dnmt1^{fl/fl}$ mice, n=9 for $Dnmt1^{KO}$ mice), or fed with 60 kcal% fat diet for 5 months before the excisional wounding (n=10). Data are presented as mean ± SEM. *$P < 0.05$ by Brown-Forsythe and Welch ANOVA followed by Holm-Sidak's post hoc test.

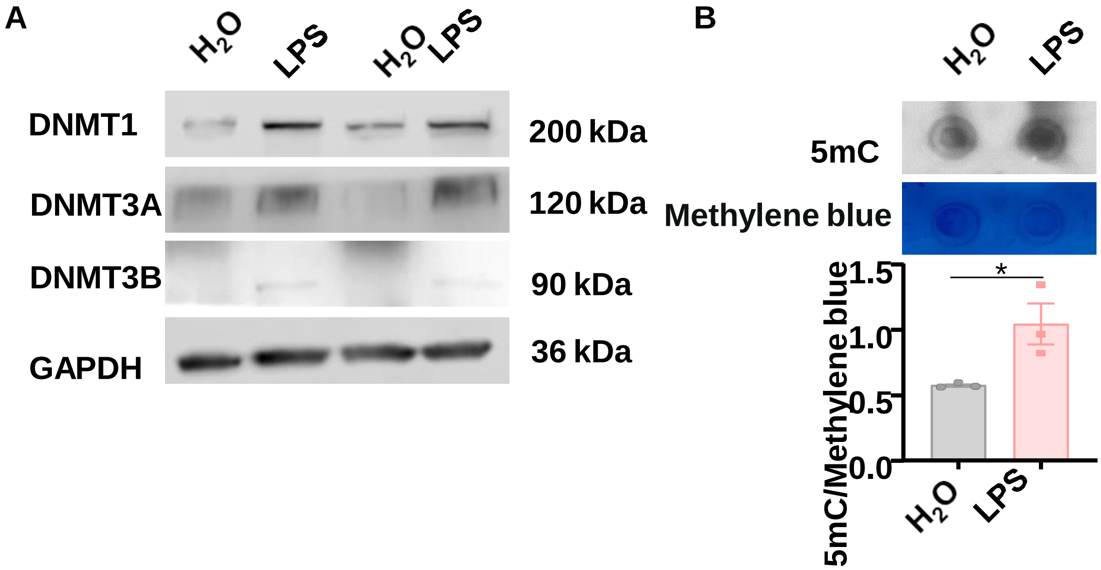

Supplementary Figure 4. LPS induces the expression of DNMTs and global methylation level in RAW 264.7 macrophages. (A) Western blotting to detect the protein level of DNMT1,DNMT3A, DNMT3B in RAW264.7 cells that were pretreated with lipopolysaccharide(LPS, 100 ng/ml) or the control H_2O for 24 h. (B) Representative images of Dot blot showing level of 5-methylcytosine in RAW264.7 cells that were pretreated with lipopolysaccharide(LPS, 100 ng/ml) or the control H_2O for 24 h. *P < 0.05 by unpaired Student's t-test.

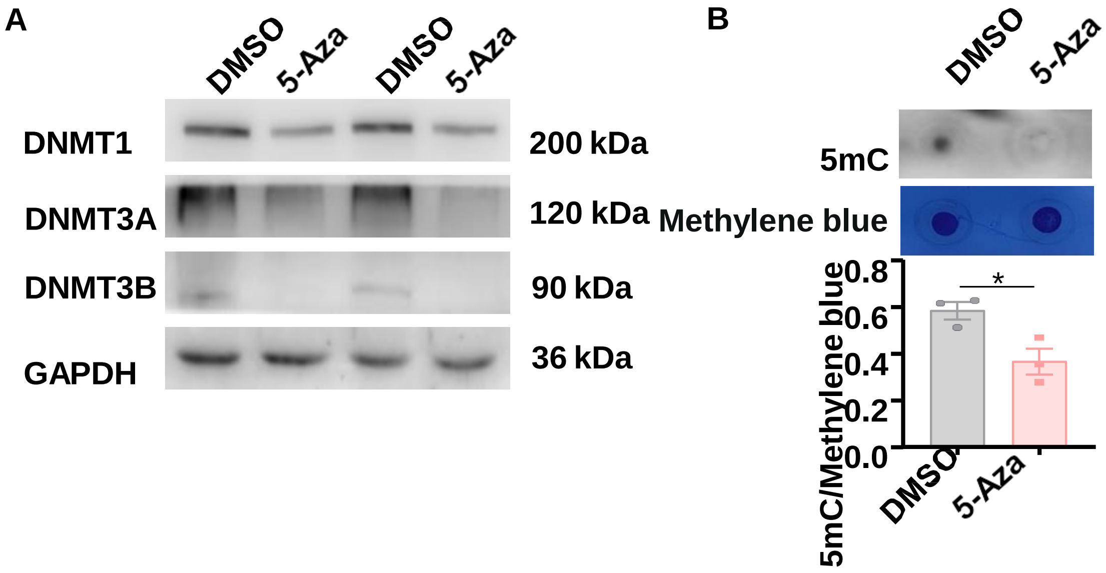

Supplementary Figure 5. 5-Aza reduces the expression of DNMTs and global methylation level in RAW 264.7 macrophages. (A) Western blotting to detect the protein level of DNMT1,DNMT3A, DNMT3B in RAW264.7 cells that were pretreated with 5-Aza (decitabine, 10 μmol/L) or DMSO for 24 h. (B) Representative images of Dot blot showing level of 5-methylcytosine in RAW264.7 cells that were pretreated with 5-Aza(decitabine, 10 μmol/L) or DMSO for 24 h. **P* < 0.05 by unpaired Student's t-test.

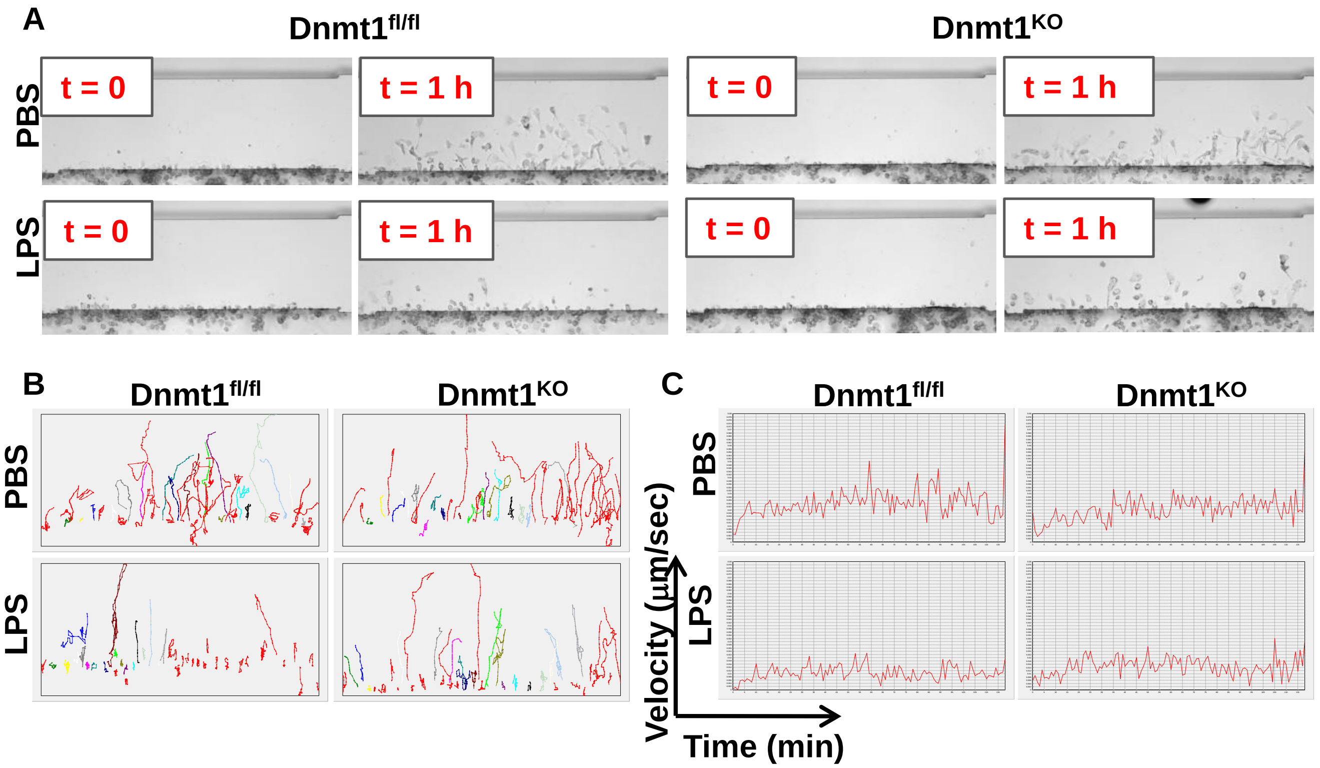

Supplementary Figure 6. Real-time horizontal chemotaxis assay to assess the motility of macrophages from the myeloid-specific Dnmt1 knockout (Dnmt1 ^{KO}) and the control (Dnmt1$^{fl/fl}$) mice. (A) Peritoneal macrophages obtained from Dnmt1^{KO} and Dnmt1$^{fl/fl}$ mice were pretreated with LPS (100 ng/ml) for 24 hours, and then subjected to a real-time horizontal chemotaxis assay (at 37 °C) toward CCL2 (20 ng/ml). The representative images taken at time 0 and 1 hour are presented. **(B)** The trajectories of all migrating cells throughout the chemotaxis assay. **(C)** Quantitation of the migration velocity of the cells.

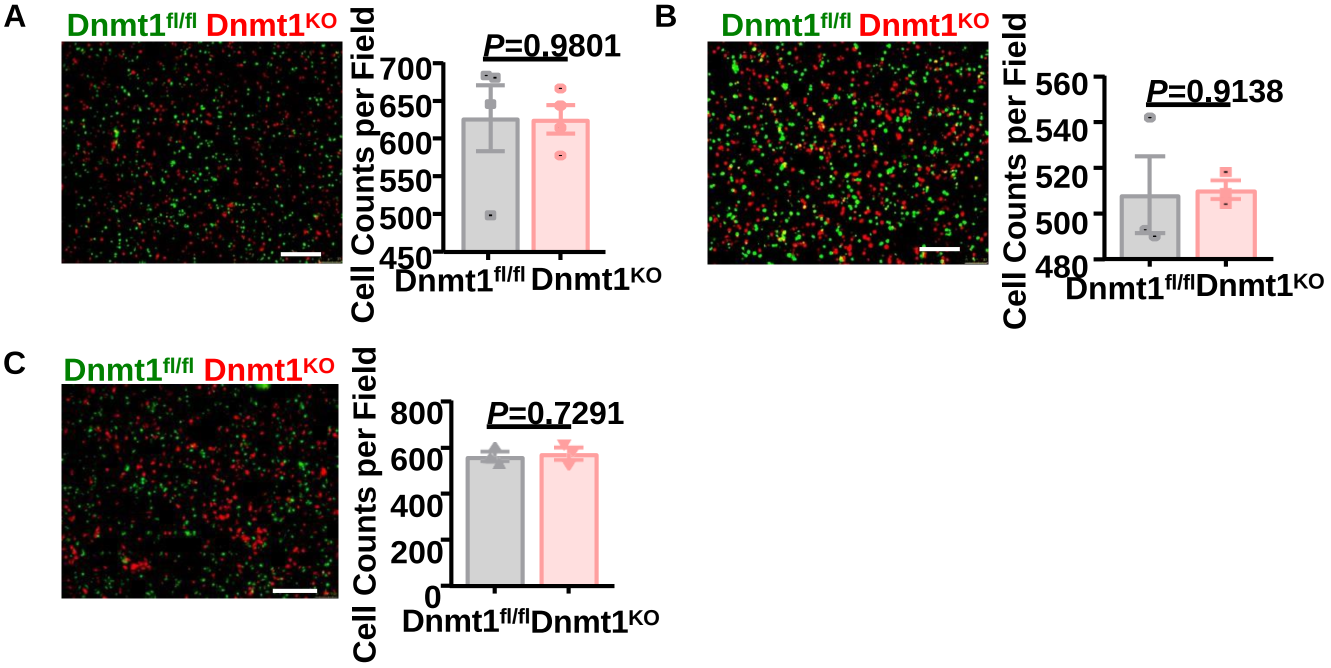

Supplementary Figure 7. Assessment of the quantity of Dnmt1$^{fl/fl}$-green and Dnmt1^{KO}-red macrophages before the in vivo migration assay. (A-C) The representative image of CM-Dil (chloromethyl-dialkylcarbocyanine, red) and DiOC$_{18}$(3) (3,3-Dioctadecyloxacarbocyanine perchlorate, green) labeled peritoneal macrophages from the Dnmt1^{KO} and the Dnmt1$^{fl/fl}$ mice. The amounts of macrophage were determined by counting the cell number in multiple fields. Scale bar, 200μm. *P* values were obtained by unpaired t-test.

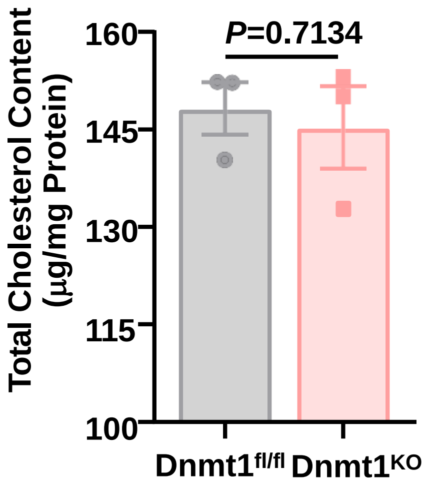

Supplementary Figure 8. The total cholesterol content in peritoneal macrophages from Dnmt1 ^{KO} and Dnmt1$^{fl/fl}$ mice. The cholesterol content were measured by an enzymatic assay kit. *P* value was obtained by unpaired t-test.

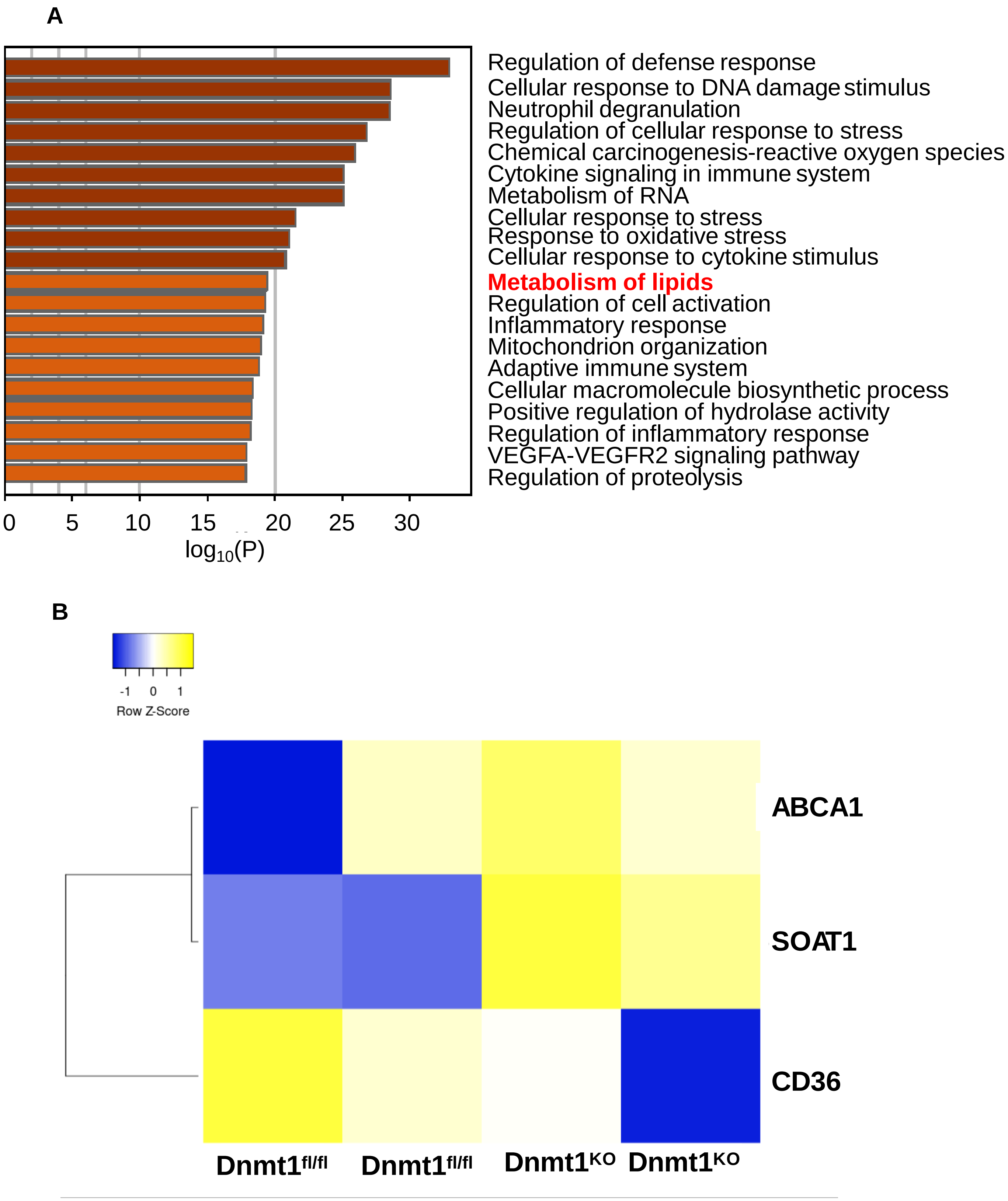

Supplementary Figure 9. The transcriptome analysis of peritoneal macrophages in the Dnmt1$^{fl/fl}$ and Dnmt1^{KO} mice. (A) A subset of genes with changes in gene expression (|$\log_2$FoldChange |≥0.2) were subjected to GO analysis. **(B)** Heatmaps of gene expression of ABCA1, SOAT1 and CD36.

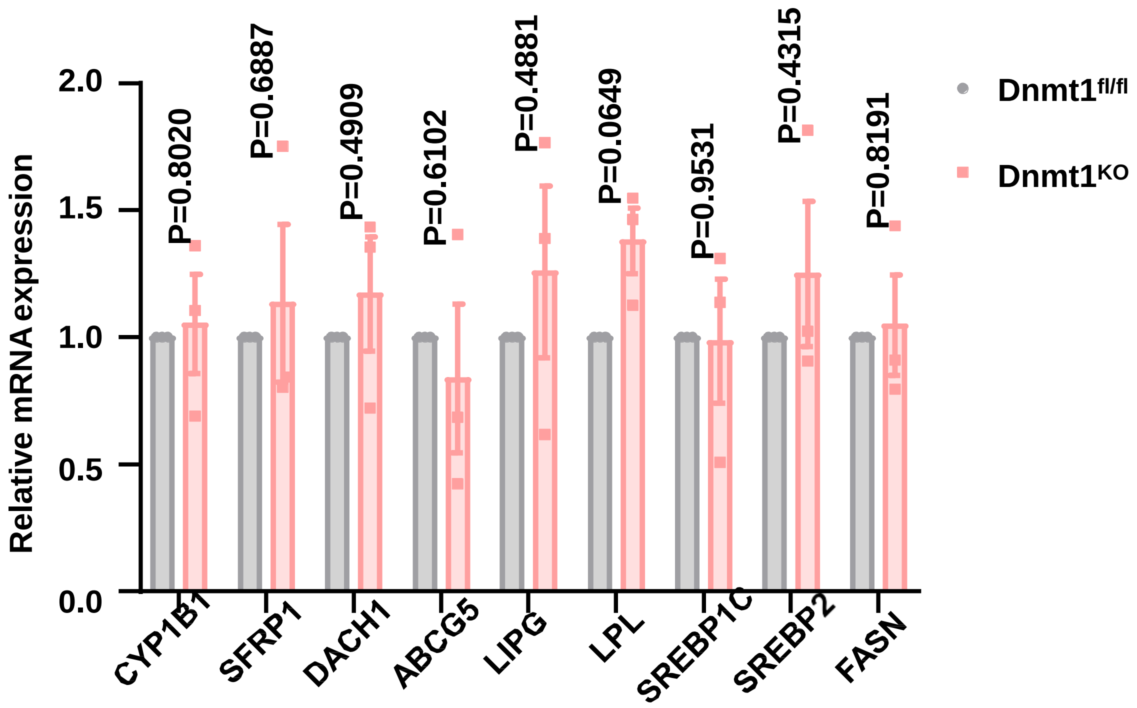

Supplementary Figure 10. Real-time qPCR analysis of expression of key genes in cholesterol synthesis in peritoneal macrophages. The cells were obtained from the $Dnmt1^{KO}$ and $Dnmt1^{fl/fl}$ mice (n = 3). Data are presented as mean ± SEM. *P* value was obtained by unpaired t-test.

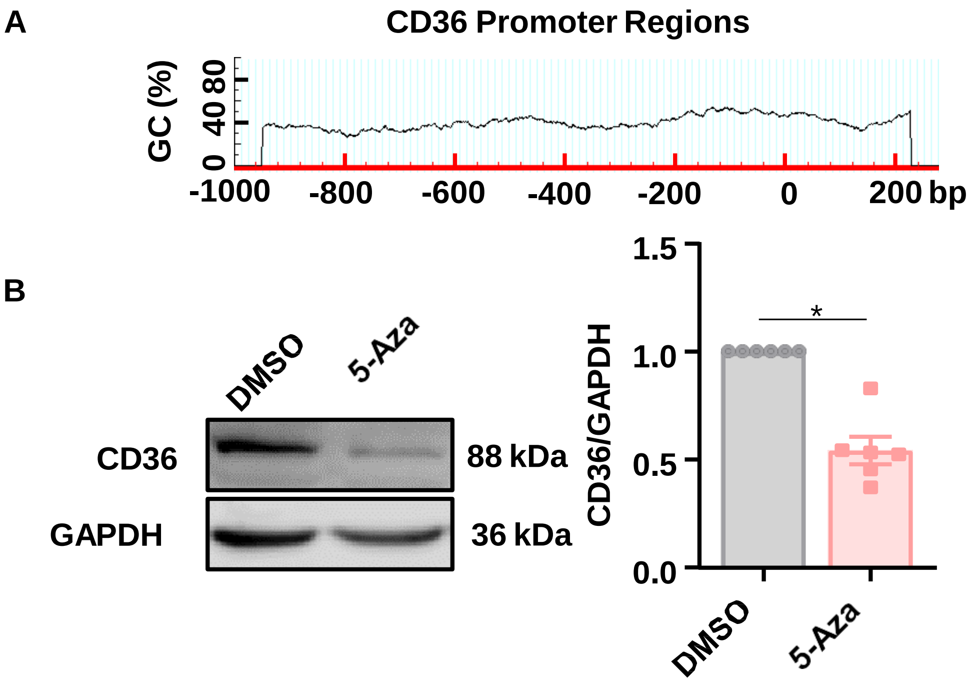

Supplementary Figure 11. The assessment of potential regulation of Dnmt1 on CD36. **(A)** GC contents in the promoter regions of CD36. **(B)** Western blotting to detect the protein level of CD36 in RAW 264.7 cells pretreated with DMSO or 5-Aza-2′-deoxycytidine (5-Aza, 10 μmol/L) . *P < 0.05 by unpaired Student‘s t-test.

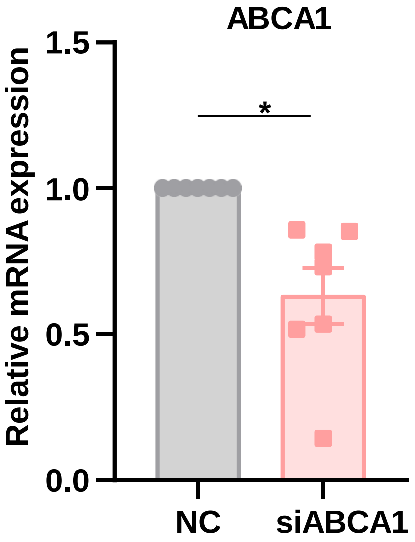

Supplementary Figure 12. Real-time qPCR analysis of expression of ABCA1 under siRNA knockdown for 48h. RAW264.7 cells were transfected with siRNA targeting ABCA1 for 48 h, gene expression was measured using real-time qPCR. Each dot representing an independent transfection experiment. Data are presented as mean ± SEM. *P < 0.05 by unpaired Student's t-test.

A

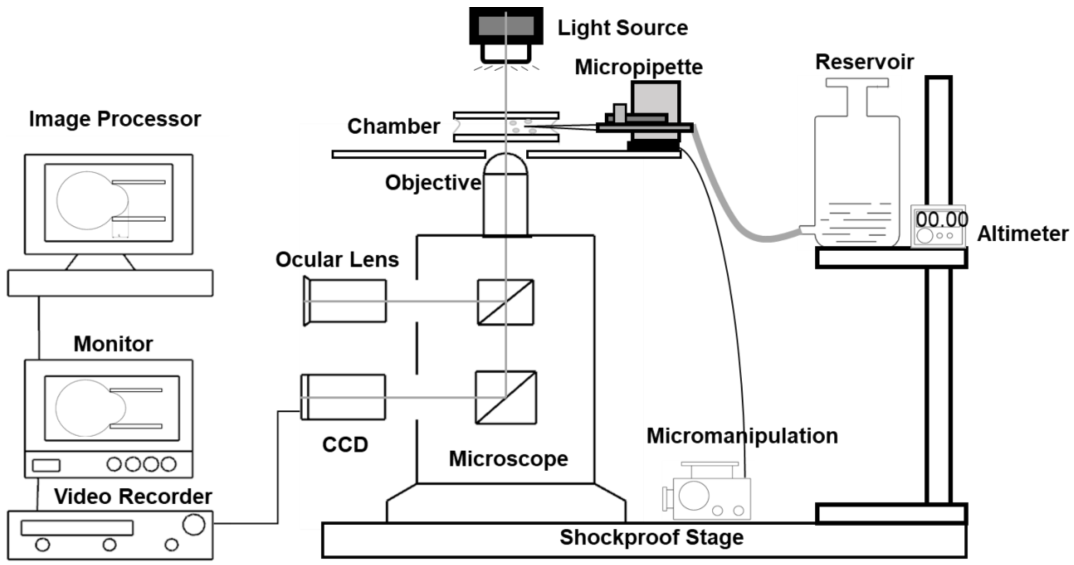

B

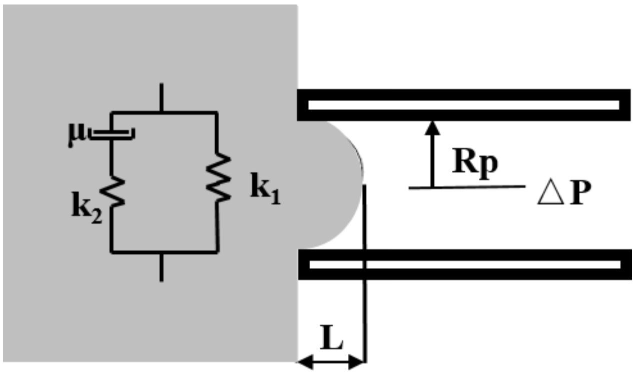

Supplementary Figure 13. Micropipette aspiration technology. (A) A schematic diagram of the micropipette aspiration technology. **(B)** A diagram of data measurement.

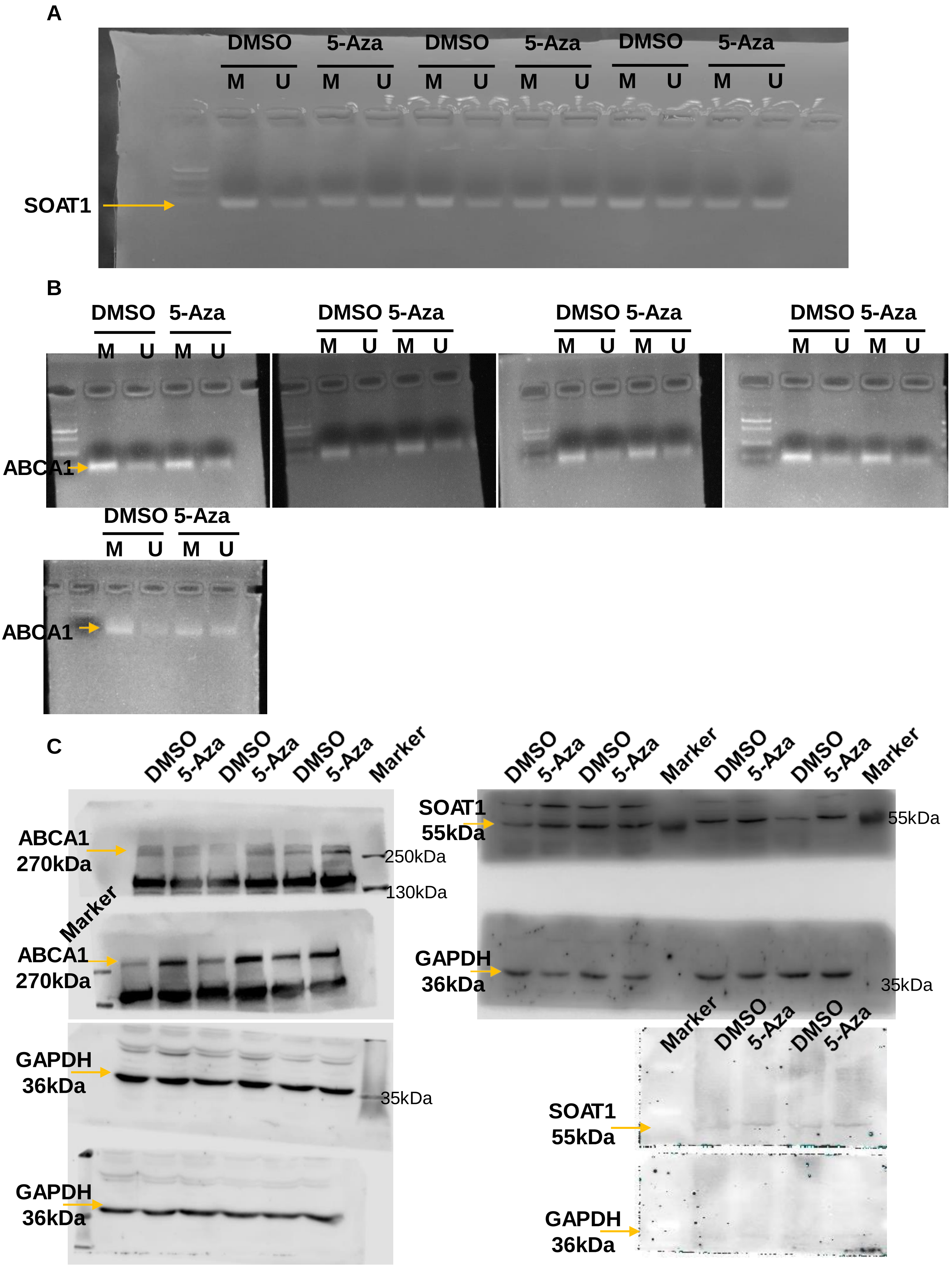

Supplementary Figure 14. Uncropped images of gels and blots for Figure 7. (A&B) MSP gel images of Figure 7C and 7D. **(C)** Uncropped images of blots of Figure 7E.

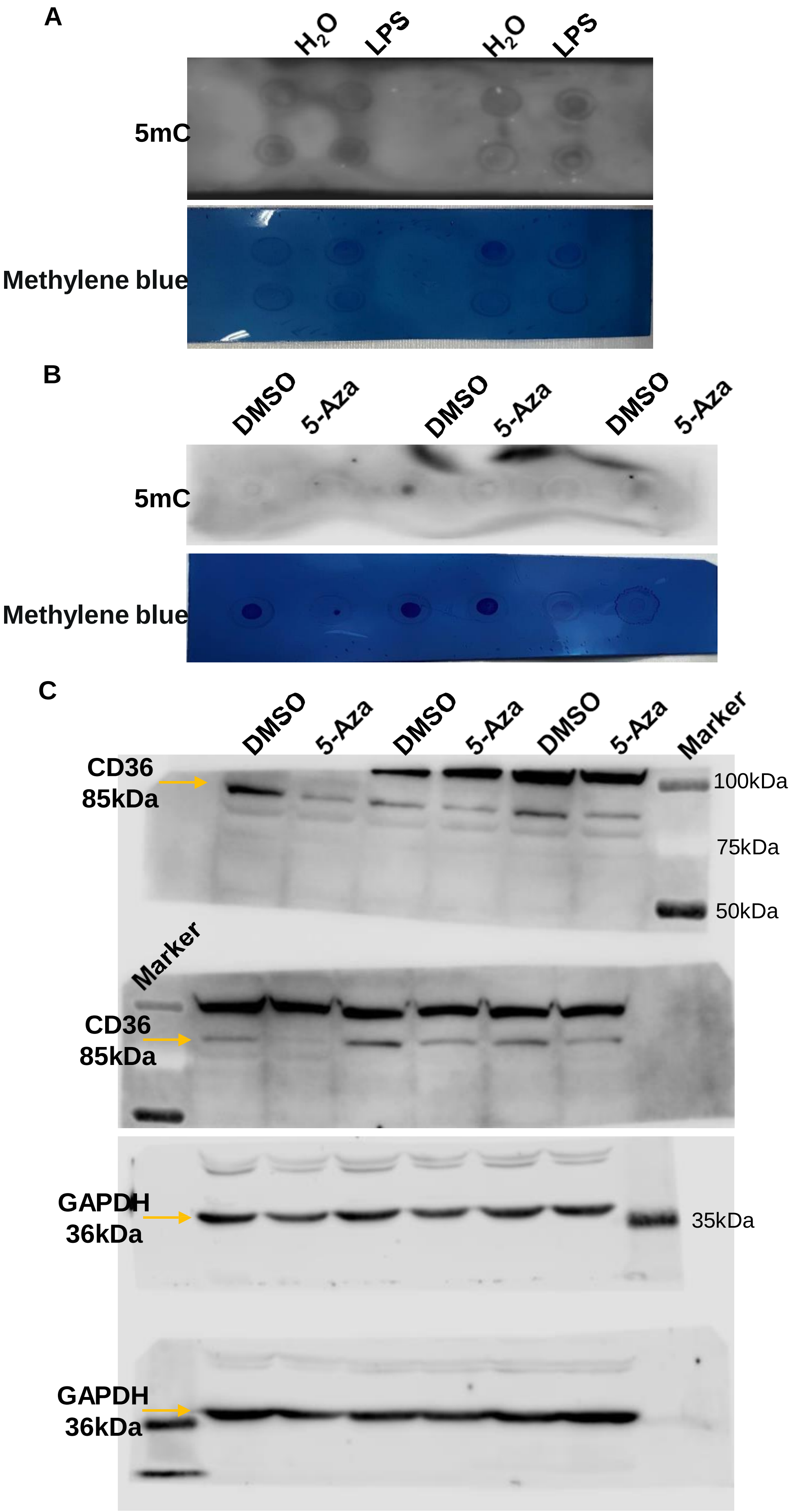

Supplementary Figure 15. Uncropped images of blots for Supplementary Figures. (A) Uncropped images of dot blots of Supplementary Figure 4B. **(B)** Uncropped images of dot blots of Supplementary Figure 5B. (C) Uncropped images of blots of Supplementary Figure 11B.

Supplementary Table

Supplementary Table 1. Primers for Qpcr, ChIP-PCR, MSP, siRNA

qPCR		
Gene name	Forward primer (5'-3')	Reverse primer (5'-3')
CD36	GCAAAGTTGCCATAATTGAGTC	GAAAGGAGGCTGCGTCTG
SR-A1	GCAGTTAAATTCCTTGATTTCGTC	GTCCTCCTGTTGCTTTGCTGTA
LOX-1	TGAAGCCTGCGAATGACGAG	GTCACTGACAACACCAGGCAGAG
SOAT1	GTCCCAACACGAAGGAGCAA	TATTAGGCAAGTGCTGTCCCC
LAL	CCGAGAAAGCAGGATCCCAA	CGCTGGAGTCGTTTGGTTCA
ACAT1	CAAGTTTAGTGGCCGGCTGA	CTGTCTTGCCAGGAAGTGGTTA
LDLR	GAAGGAGAGGAAGCCCAAACC	CTCTATTCCCAACCCCCACTC
NCEH1	GTGCACAACAATGGCTGAGG	GAGAGTTGTGACTAGTGGTAGC
SR-BI	GCCCATCATCTGCCAACT	TCCTGGGAGCCCTTTTTACT
ABCG1	GTCTGAACTGCCCTACCTAC	GGTCTCTCTTATAGTCAGCG
ABCA1	TGGGCTCCTCCCTGTTTTTG	CTCTGAGAAACACTGTCCTCCTTT
GAPDH	GGACCTCATGGCCTACATGG	TAGGGCCTCTCTTGCTCAGT
DNMT1	CTCGGGCCAATCAATCAGTG	GCTCATACTCTTTGCTGGGC
ChIP-qPCR		
Gene name	Forward primer (5'-3')	Reverse primer (5'-3')
SOAT1	TCCTAGTCTCCGACCGTCC	CTTCTCTTCTCCCACTATC
ABCA1	GACTCAAACAGCAAAGTGG	GTCACCACAGCCGGCACCC
MSP		
Gene name	Forward primer (5'-3')	Reverse primer (5'-3')
MSP Abca1 Methylated	GTTAGGGTTAGGGTTATAGAAAGCG	CAATCACTCAACAAAAAACACGTA
MSP Abca1 Unmethylated	TTAGGGTTAGGGTTATAGAAAGTGG	TCAATCACTCAACAAAAAACACATA
MSP Soat1 Methylated	CGTAGGATTATTAATGGTATTTCGG	AAACAACTACGACGATCTAAACGAT
MSP Soat1 Unmethylated	TGTAGGATTATTAATGGTATTTTGG	AAACAACTACAACAATCTAAACAAT
siRNA		
Gene name	Forward primer (5'-3')	Reverse primer (5'-3')
siABCA1	GUGUCUACGUGCAACAGAU	AUCUGUUGCACGUAGACAC
